# Low-Frequency Raman Spectroscopy of Pure and Cocrystallized Mycophenolic Acid

**DOI:** 10.3390/pharmaceutics15071924

**Published:** 2023-07-11

**Authors:** Catherine S. Wallace, Margaret P. Davis, Timothy M. Korter

**Affiliations:** Department of Chemistry, Syracuse University, 1-133 Center for Science and Technology, Syracuse, NY 13244-4100, USA; cswallac@syr.edu (C.S.W.); mdavis23@syr.edu (M.P.D.)

**Keywords:** vibrational spectroscopy, terahertz vibrations, periodic boundary conditions, crystal engineering

## Abstract

The aqueous solubility of solid-state pharmaceuticals can often be enhanced by cocrystallization with a coformer to create a binary cocrystal with preferred physical properties. Greater understanding of the internal and external forces that dictate molecular structure and intermolecular packing arrangements enables more efficient design of new cocrystals. Low-frequency (sub-200 cm^−1^) Raman spectroscopy experiments and solid-state density functional theory simulations have been utilized together to investigate the crystal lattice vibrations of mycophenolic acid, an immunosuppressive drug, in its pure form and as a cocrystal with 2,2′-dipyridylamine. The lattice vibrations primarily consist of large-amplitude translations and rotations of the crystal components, thereby providing insights into the critical intermolecular forces governing cohesion of the molecular solids. The simulations reveal that despite mycophenolic acid having a significantly unfavorable conformation in the cocrystal as compared to the pure solid, the cocrystal exhibits greater thermodynamic stability over a wide temperature range. The energetic penalty due to the conformational strain is more than compensated for by the strong intermolecular forces between the drug and 2,2′-dipyridylamine. Quantifying the balance of internal and external energy factors in cocrystal formation indicates a path forward in the development of future mycophenolic acid cocrystals.

## 1. Introduction

Formation of pharmaceutical cocrystals is a standard method for improving the solubility of active pharmaceutical ingredients (APIs) in solid-state formulations which can then enhance drug bioavailability [1]. Defined as a crystalline material composed of two or more uncharged components with stoichiometric ratios, the unique three-dimensional packing arrangements found in a cocrystal differ from those of the pure ingredients. These changes lead to modified physical properties [2]. Great diversity exists in pharmaceutical cocrystals as researchers continuously work to progress solid-state drug efficacies, but they are often composed of one API and one biologically inactive organic molecule. Numerous design schemes exist for creating new cocrystalline solids; however, detailed knowledge of both the intermolecular and intramolecular forces between and within the crystal components is foundational to rational approaches in crystal engineering [3]. The crystal structures and the key energetic factors can be understood using various characterization methods.

The most common methods of cocrystal characterization include powder and single-crystal X-ray diffraction (PXRD and SCXRD), solid-state nuclear magnetic resonance (NMR) spectroscopy, and vibrational spectroscopy (infrared (IR) and Raman) [4,5]. SCXRD provides atomic-level information about a cocrystal’s solid-state structure, while PXRD is most often used to determine crystal lattice parameters for bulk microcrystalline samples. Solid-state NMR similarly studies the three-dimensional structure of a crystal at the atomic level and has been used alongside SCXRD for characterizing pharmaceutical cocrystals [6]. Vibrational spectroscopy can be particularly useful in cocrystals as substituent vibrations occurring in the group frequency region are sensitive to the degree of hydrogen bonding [5].

To expand their solid-state characterization abilities, both IR and Raman spectroscopies have been extended to lower vibrational frequencies so that crystal lattice vibrations existing in the 10 to 200 cm^−1^ (0.3 to 6.0 THz) range can be readily measured. Terahertz or far-IR spectroscopy has been demonstrated in numerous studies as a reliable technique for characterizing crystalline pharmaceuticals [7,8]. Low-frequency Raman spectroscopy (LFRS) complements terahertz spectroscopy with similar spectral coverage and has gained recent attention due to technological advances making instruments available with better performance and lower cost [9,10]. The lattice vibrations (e.g., translations and torsions) probed by LFRS are unique to the chemical identities and crystal packing within samples, directly reflecting the intermolecular and intramolecular forces at play within the cocrystal. This distinctiveness is advantageous for establishing the definitive nature of an LFRS signature, but it also often complicates data interpretation and necessitates detailed computational analyses.

Computational methods are required to fully assign the LFRS spectra to specific atomic motions and understand how these vibrations then correlate with either interactions between molecules or energy factors internal to the molecules. Solid-state density functional theory (ss-DFT) simulations enable the lattice motions of the crystalline solid to be modeled with high accuracy [11]. The ability of ss-DFT to reproduce experimental LFRS observations is specifically indicative of the intermolecular forces being well modeled, as these strongly influence both internal and external vibrational frequencies and intensities. Confidence in the ss-DFT models earned from the successful reproduction of LFRS data indicates confidence can also be had in the conformational and cohesive energy calculations of the solid, which is particularly valuable for monitoring changes between related systems [12,13]. Collectively, the high-quality energies and vibrational frequencies can be used to construct reliable Gibbs free energy curves to provide insights concerning the thermodynamic stabilities of cocrystalline species.

**Figure 1:**
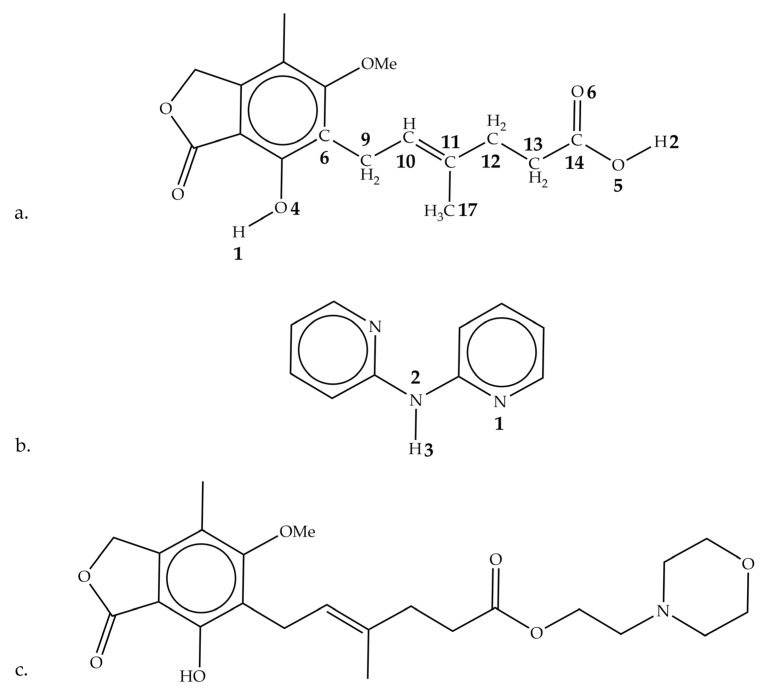
Structural formulas for (**a**) MPA, (**b**) DPA, and (**c**) MMF.

In this work, the immunosuppressive drug mycophenolic acid (MPA) is studied in both its pure form and as a cocrystal with 2,2′-dipyridylamine (DPA) [14,15]. Best known for its use in preventing organ transplant rejection, MPA is commonly administered in its solid-state form as either a sodium salt (Myfortic™) or in an ester form prodrug as mycophenolate mofetil (MMF, CellCept™). Structural formulas of these compounds are provided in Figure 1. Atom numbering is consistent with previous work [15]. However, modifications of MPA continue to be studied with the aforementioned goal of improving physical properties but also to address issues such as its gastrointestinal side effects [14,15]. The DPA molecule is a heterocyclic nitrogen-containing compound that serves as an organic base and is often utilized as a ligand in coordination chemistry and coformer in cocrystal engineering [16]. The crystal structure of the MPA:DPA cocrystal was recently reported and serves as the focus of this work [15]. The sub-200 cm^−1^ Raman spectra of the pure and cocrystalline species exhibit numerous distinct features that can be understood through the application of ss-DFT simulations in the analysis of each molecular solid. The calculations reveal that the cocrystal has significant thermodynamic stability over a range of temperatures, which can be linked to the relative cohesive and conformational energies within the solids. The results of the MPA:DPA study are then compared with the commercially available pharmaceutical solid MMF to compare and contrast the energy differences in the solid-state forms of MPA derivatives.

## 2. Materials and Methods

### 2.1. Sample Preparation

Mycophenolic acid (98%, C_17_H_20_O_6_, CAS #: 24280-93-1) and 2,2′-dipyridylamine (>99.0%, C_10_H_9_N_3_, CAS #: 1202-34-2) were purchased through Fisher Scientific (Hampton, NH, USA) and used without further purification. Following the previously published procedure, the 1:1 stoichiometric ratio of MPA:DPA cocrystal was grown by slow evaporation at ambient laboratory conditions of 1:1 molar ratio solutions (0.01 M) of the compounds in ethyl acetate [15]. For long term storage (>1 day), MPA was kept in a freezer at 255 K, while DPA was kept under ambient laboratory conditions. MPA:DPA cocrystal samples were stored under ambient laboratory conditions. All samples were analyzed using PXRD at room temperature with a Bruker (Billerica, MA, USA) D2 Phaser diffractometer (Cu K_α_ radiation, λ = 1.54060 Å, 2Θ = 5 to 70° with 0.5 s per 0.02° step). The crystal structures were confirmed through comparison to PXRD patterns calculated from experimental data (see Supplementary Materials) available in the Cambridge Structural Database (CSD) (Cambridge, UK) [17].

### 2.2. Low-Frequency Raman Spectroscopy (LFRS)

Raman spectra of MPA, DPA, and MPA:DPA were measured using a modified Coherent (Santa Clara, CA, USA) THz-Raman bench instrument with 784.7 nm laser excitation and a power of 100 mW with a laser spot size of approximately 0.5 mm to avoid sample damage. Raman scattered radiation was analyzed using an Andor (Belfast, UK) Shamrock SR-750 spectrograph equipped with a cooled iDus 416 CCD camera. For data collection, 225 acquisitions were taken with 3 s exposure time windows (~15 min in real time), yielding spectra with a range of 10 to 300 cm^−1^ and 0.6 cm^−1^ spectral resolution. Powdered microcrystalline samples of the pure and cocrystal solids were prepared through mild grinding of the material with a mortar and pestle to reduce crystallite inhomogeneity. The prepared samples were then mounted in a Janis (Westerville, OH, USA) ST-100 optical cryostat with a customized brass cuvette system and transparent glass windows. Experiments were conducted at both laboratory (295 K) and liquid-nitrogen (78 K) temperatures. Data were processed using Spectragryph v1.2.16.1 [18] (Oberstdorf, Germany) for baseline correction and the removal of interference peaks from the rotational spectra of atmospheric N_2_ and O_2_.

### 2.3. Computational Methods

Solid-state density functional theory simulations were run using periodic boundary conditions with the CRYSTAL17 commercial software package (Torino, Italy) [19]. The Perdew–Burke–Ernzhof [20] (PBE) density functional was used in conjunction with Ahlrichs’ VTZ [21] basis set with added polarization functions [22]. Grimme’s London dispersion correction (D3) with Becke-Johnson damping [23,24,25] and the three-body Axilrod-Teller-Muto [26] (keyword: ABC) correction supplemented these calculations. This general approach has been successfully applied in previous investigations of molecular solids [12,27]. An integration grid consisting of 99 radial points and 1454 angular points was used for all calculations (keyword: XXLGRID). The overlap integration thresholds for the Coulomb and exchange bielectronc integrals were set to 10^−10^ and 10^−20^ (keyword: TOLINTEG 10 10 10 10 20). All calculations were Γ-point only, and phonon dispersion effects were not considered.

Geometry optimizations of each sample were performed using experimental CSD data as the starting crystal structure and run until an energy convergence of ΔE < 10^−8^ Hartree (10^−5^ kJ/mol) was achieved. Normal mode vibrational frequency analyses were performed on the optimized structures using numerical derivatives computed by the central-difference formula, with two displacements per atom in each of the Cartesian directions. The normal mode analyses were done using a stricter energy convergence of ΔE < 10^−10^ Hartree (10^−7^ kJ/mol). IR and Raman intensities of the vibrational modes were calculated using the coupled-perturbed Hartree–Fock/Kohn–Sham approach (keyword: INTCPHF) [28,29]. Experimental parameters of sample temperature and excitation laser wavelength were also applied in the calculation of Raman intensities presented in the final simulated spectra [30,31].

Cohesive and conformational energy calculations were performed with an energy convergence of ΔE < 10^−8^ Hartree (10^−5^ kJ/mol). The total energy of each unit cell and the conformational energies of each symmetry-unique molecule (keyword: MOLECULE) were calculated based on the ss-DFT optimized geometries. Subtracting the summation of the molecular conformational energies from the respective total unit cell energy yielded the cohesive energy of the crystal structure. The cohesive energy values were corrected for basis-set superposition error (BSSE) using the counterpoise method (keyword: MOLEBSSE). The distance (up to 10 Å) and number of atoms (up to 240) considered for each energy calculation were incrementally increased until a BSSE correction convergence of ΔE < 0.5 kJ/mol (per molecule) was reached for each studied solid.

Single-molecule calculations were also performed using CRYSTAL17 with the same computational methodology as for the solid state. 

## 3. Results and Discussion

### 3.1. Crystal Structure Analyses

The ss-DFT optimizations of the pure crystal structures of MPA and DPA as well as the cocrystalline MPA:DPA were started from their initial experimentally determined positions, utilizing the space group symmetries of each structure. The crystallographic information available in the CSD indicated a symmetry of $P\bar{1}$ for MPA (Z = 2, Refcode: MYCPHA02), Pccn for DPA (Z = 8, Refcode: DPYRAM), and $P2_{1}/c$ for MPA:DPA (Z = 4, Refcode: XAVTEW). After optimization, percent error values for each of the lattice dimensions and root-mean-square deviation (RMSD) values for bond lengths, bond angles, and torsional angles were calculated to quantify the accuracy of the simulations. Collectively, the unsigned average percent error for the lattice dimensions of MPA, DPA, and MPA:DPA was less than 1.5% (Table 1). The RMSD values for each of the structures, considering only non-hydrogen atoms, were similar to each other (Table 2). Unexpectedly, the larger errors in the torsional angles of the MPA and MPA:DPA crystals can mainly be attributed to the dihedral angle of the methoxy group in MPA, although the flexibility of the hydrocarbon chain present in MPA also leads to increased sensitivity of its torsional angles to the calculated intermolecular forces.

The geometric evaluation of the intermolecular forces in each crystal structure focused on quantification of the hydrogen bonding arrangements of the crystal components (Table 3). Crystalline MPA packing is guided by end-to-end hydrogen bonding between the carboxylic acid tails of two neighboring MPA molecules, as well as interactions between one molecule’s carboxylic acid tail and another’s aromatic ring hydroxyl group. For DPA, the amine group is the only hydrogen donor site available for hydrogen bonding, and the pyridyl nitrogen serves as an acceptor, yielding hydrogen bonded DPA dimers in the solid. Similar patterns are present in the cocrystal with the largest change being the carboxylic acid of MPA interacting with the amine group in DPA, replacing the DPA dimeric interaction. It is noteworthy that the conformation of the MPA molecule changes significantly between the pure crystal and the cocrystal to achieve this hydrogen bonding arrangement, and this will be considered in the discussion of the crystal energies. An RMSD evaluation of only the heavy-atom distances in the hydrogen bonds was considered due to the relative inaccuracy of the experimental hydrogen positions. These values were 0.08 Å, 0.14 Å, and 0.04 Å for MPA, DPA, and MPA:DPA, respectively, showing good correlation with the experiments and correctly following the experimental trends for longest and shortest hydrogen bonds across the solids. With the ss-DFT optimized structures showing low errors in both the intramolecular and intermolecular geometries, the accuracy of the simulations is reasonable for the next steps in elucidating these structures using normal mode vibrational analyses.

### 3.2. Raman Spectroscopy and Peak Assignments

**Figure 2:**
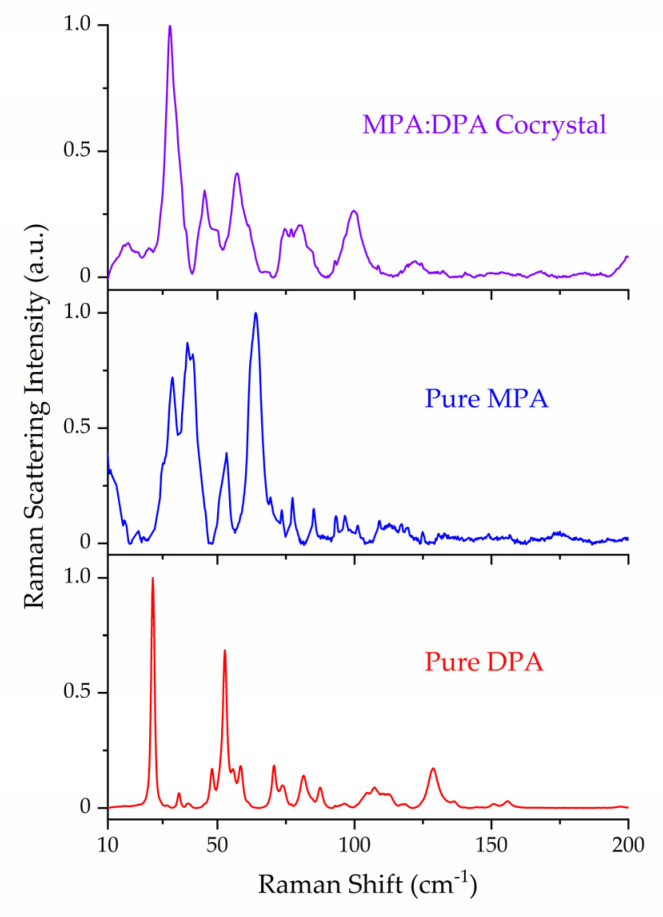
Experimental Raman spectra of DPA (red), MPA (blue), and MPA:DPA (purple) at 78 K. Spectral intensities have been normalized to 1.0 within each data set.

**Figure 3:**
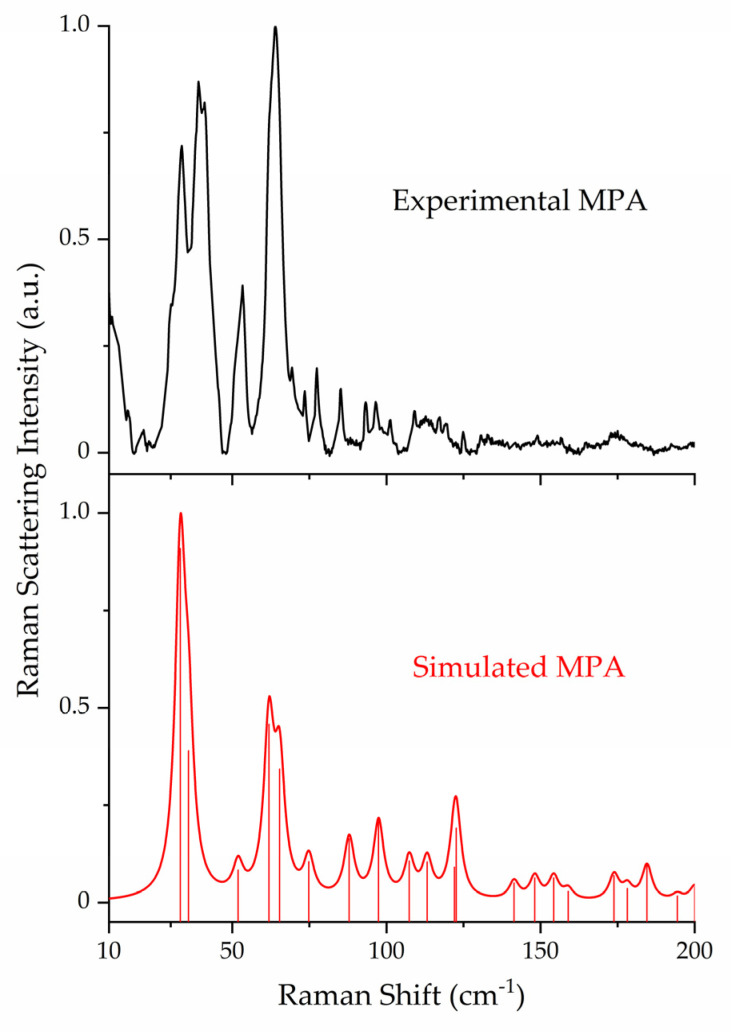
Comparison of experimental (78 K, black) and simulated (red) Raman spectra of MPA. Spectral intensities have been normalized to 1.0 within each data set.

**Figure 4:**
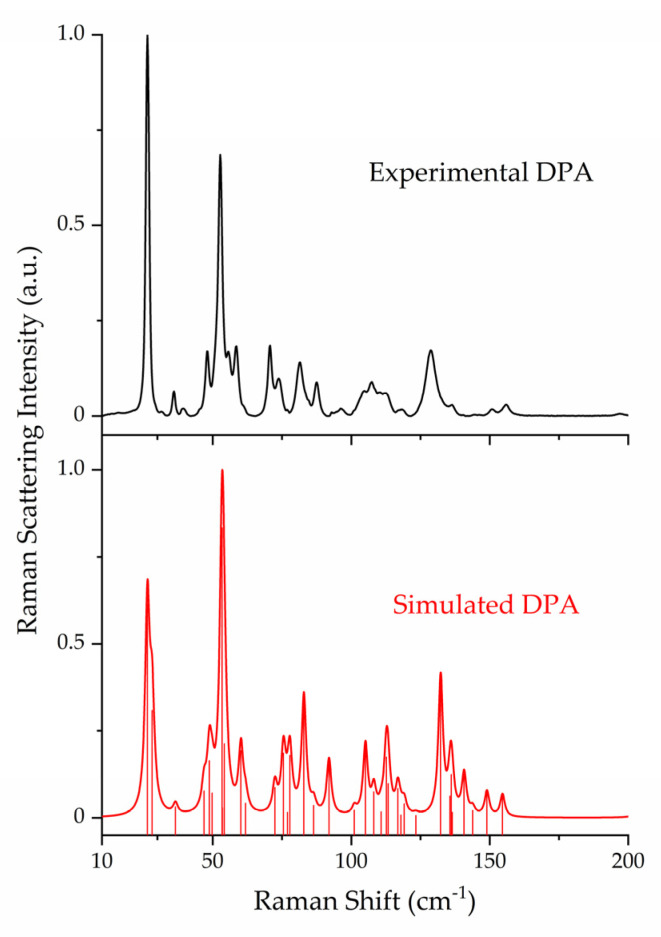
Comparison of experimental (78 K, black) and simulated (red) Raman spectra of DPA. Spectral intensities have been normalized to 1.0 within each data set.

**Figure 5:**
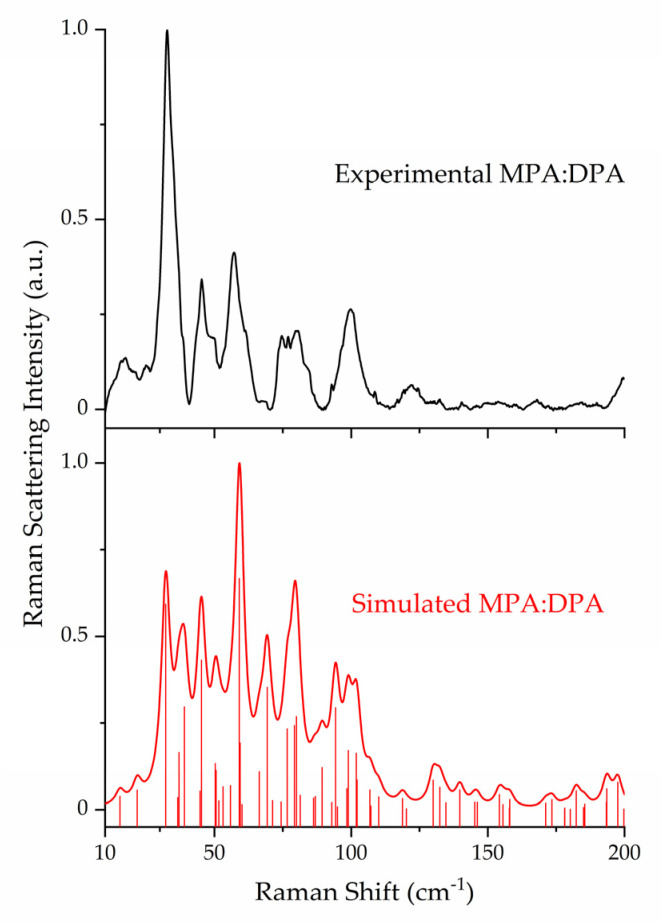
Comparison of experimental (78 K, black) and simulated (red) Raman spectra of MPA:DPA. Spectral intensities have been normalized to 1.0 within each data set.

The ss-DFT normal mode frequency analyses of the MPA, DPA, and MPA:DPA structurally optimized crystals yielded no imaginary frequencies, confirming that the structures corresponded to potential energy surface minima. Complete listings of all vibrational frequencies and intensities are available in the Supplementary Materials. Comparison of the experimental Raman data of the API, coformer, and cocrystal highlights the uniqueness of the sub-200 cm^−1^ spectral features and underlying vibrations (Figure 2). The lattice vibrations of each compound are distinct from one another due to the unique packing within the crystal structures which is a direct consequence of the intermolecular and intramolecular forces present in the solids. The experimental and simulated Raman spectra of all three crystals are shown in Figure 3, Figure 4 and Figure 5. For aid in comparison to experiment, an empirical Lorentzian line shape with a full-width-at-half-maximum (FWHM) of 2 cm^−1^ has been added to the MPA and MPA:DPA spectra, while a FWHM of 1 cm^−1^ was used for the DPA spectrum. Visual comparisons of the simulated spectra to the experimental spectra show good reproductions of the spectral peak positions and intensities. A seemingly poor reproduction of the MPA:DPA spectrum is seen in Figure 5. However, the mismatch is amplified by the much higher density of vibrational states in the cocrystal spectrum compared to the pure MPA and DPA spectra. The large number of MPA:DPA cocrystal vibrations in this region creates a situation where even small peak shifting in the simulated spectrum results in large changes in the peak shape overlap between neighboring features. This ultimately results in the MPA:DPA vibrational simulation being highly sensitive to even small errors in the simulation, leading to what appears to be larger errors than the pure substances.

In general, the descriptions of normal modes of vibrations in molecular crystals can be classified in two ways, internal motions such as torsional vibrations and external motions such as translations and rotations. It is important to note that external motions are dominated by strong intermolecular hydrogen bonds in the solids considered here, and the large-amplitude motions preserve these interactions. Multiple occurrences of the same basic type of vibrational motion exist in these solids (and appear in the mode character descriptions) due to the different molecules in the Z > 1 crystallographic unit cells undergoing phase-related movements based on the crystal symmetry. Assignments of the Raman features to specific atomic motions are provided in Table 4 and Table 5. Only spectral peaks with significant signal-noise ratios were assigned, specifically those with intensities above 0.10 for MPA and 0.05 for both DPA and MPA:DPA. The assignments of simulated peaks to experimental peaks were driven by both frequency positions and relative Raman scattering strengths, rather than by frequency matching alone.

Not all crystalline solids will have equal contributions in their Raman spectra from internal and external motions. As listed in Table 4, MPA exhibited more internal motions whereas DPA had more external motions in the sub-200 cm^−1^ region. This is in part due to the flexible carbon chain present in MPA that allows for more torsional vibrations. Relevant to this point, the first intense peak at 33.6 cm^−1^ in the pure MPA spectrum corresponds to ring-chain torsion, and the most intense peak at 63.8 cm^−1^ is a combination of two ring-chain torsions. For DPA, the most intense peak at 26.4 cm^−1^ is composed of two nearly degenerate external vibrations, one rotation and one translation. Considering the internal motions of DPA, all in this spectral region were torsional and centered on the amine linkage between the pyridine rings.

The important Raman-active vibrations of the MPA:DPA cocrystal are listed in Table 5. The first and most intense peak in the MPA:DPA spectrum at 32.7 cm^−1^ is composed of three separate rotations with atomic displacements that preserve the O-H···N and N-H···O hydrogen bonding scheme. The broader peaks (broader due to the overlap of numerous vibrations of similar frequency) around 75–85 cm^−1^ correspond to more torsions within MPA, along the chain and of the methoxy group on the ring, while those around 95–105 cm^−1^ are due to ring-ring torsions localized to DPA. Like the other low-frequency lattice vibrations of the cocrystal, as the DPA rings twist, the carboxylic acid group on MPA follows to maintain a favorable hydrogen bond geometry. The ring-chain torsions that were prevalent in the pure MPA crystal are less pronounced in the cocrystal for similar reasons involving the intermolecular hydrogen bonds.

### 3.3. Evaluation of Conformational and Cohesive Energies

**Figure 6:**
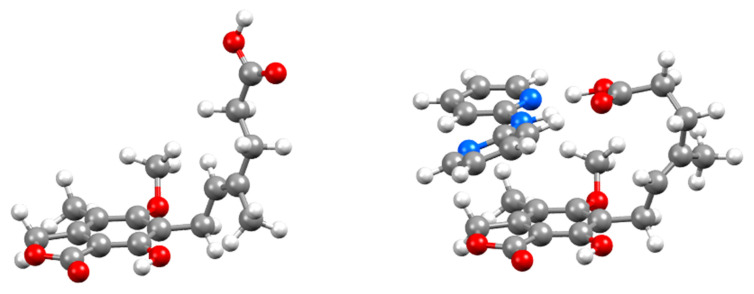
Conformation of MPA in the pure crystalline form (**left**) and in the MPA:DPA crystallographic asymmetric unit (**right**). Atom colors: red = O, blue = N, gray = C, and white = H.

Calculation and comparison of the conformational and cohesive energies of MPA and DPA in their pure crystal forms and in the cocrystal allow for further understanding of the formation and stability of MPA:DPA. The results of the ss-DFT crystal structure optimizations are considered here first. As noted earlier, MPA has substantial conformational flexibility in the carbon chain. This manifests as large rotations of the carboxylic acid tail of MPA when in the cocrystal (Figure 6). In the pure conformation, MPA has dihedral angles of 117.2°, 176.2°, and −176.8° between C6-C9-C10-C11, C11-C12-C13-C14, and C17-C11-C12-C13, respectively, in the chain. When coformed with DPA, the conformation of MPA then adopts dihedral angles of −130.5°, 60.8°, and 76.1° for the same carbons. These torsional angles reveal that large changes of up to 115° for each dihedral are occurring upon cocrystallization. Single-point energy calculations of MPA show that it has considerable conformational strain in the cocrystalline solid, with a molecular energy that is 17.83 kJ/mol greater than in its pure form.

Conversely, DPA shows a moderate conformational energy improvement in part of the cocrystal, where its conformation is 2.84 kJ/mol lower in energy than DPA in its pure form. The opposite relationship is found for the cohesive energies. The MPA pure crystal exhibits a cohesive energy per atom of −4.76 kJ/mol per atom, while pure DPA is −5.62 kJ/mol per atom. In the combined solid, the MPA:DPA cocrystal has a cohesive energy of −5.42 kJ/mol per atom, greater in magnitude than MPA and less than DPA. This is consistent with the very large conformational energy increase of MPA upon cocrystallization being countered by a strengthening of the cohesive energy in MPA:DPA. Likewise, DPA shows a similar behavior but instead has a small improvement in conformational energy that is balanced by a small degrading of the cohesion. Overall, this indicates that the less favorable molecular conformation that MPA is able to adopt in the process of cocrystal formation is the key event in establishing new stabilizing intermolecular interactions, thereby yielding a stable MPA:DPA cocrystal.

The difference in the MPA conformational energy between the pure and cocrystalline solids is significant and worthy of further consideration, even in terms of an isolated MPA molecule. The conformational energy of a molecule in the solid state is not expected to be the same as an isolated (gas phase) molecule or one in solution. To explore this, single-molecule optimizations of MPA and DPA were achieved in the gas phase, starting from both the pure crystal conformations and the cocrystal conformations of each. A full conformational analysis of each molecule is not presented here due to the focus being on simple comparisons of the crystallized conformations to the conformations of the solid-state molecules allowed to relax in isolation, but a full conformational analysis of MPA has been reported previously [33]. As anticipated, the flexible MPA molecule relaxed the most upon free optimization. While both DPA starting points converged to a single structure after optimization, the two MPA optimizations produced two unique geometries, referred to here as MPA(pure) and MPA(cocrystal). Upon relaxation, there were no large changes in particular dihedral angles, but numerous smaller changes occurred throughout the torsional angles defining the chain geometry. Frequency analyses were run on each optimized molecule and confirmed all were at energetic minima. The two MPA structures had distinct energy differences with the cocrystalline conformation of MPA leading to a gas-phase optimized structure 6.52 kJ/mol lower in energy than that started from the pure crystal geometry. For both MPA and DPA, the single-molecule optimizations significantly lowered the conformational energies of the molecules compared to the solid conformations. Relative to the single-point energies of the rigid molecules extracted from the crystals, the gas-phase optimization lowered the conformational energy of MPA(pure) by 17.09 kJ/mol and MPA(cocrystal) by 41.43 kJ/mol. This result emphasizes the considerable conformational strain the crystallized MPA molecule has, especially when incorporated into the MPA:DPA cocrystal as it sacrifices internal energy to promote external noncovalent bonding. For DPA, the isolated molecule results are similar to MPA, but the energy changes are smaller given the relatively minor changes observed in its molecular shape. The gas-phase optimization of DPA(pure) relaxed the molecule by 10.75 kJ/mol, and DPA(cocrystal) achieved a 7.90 kJ/mol lower energy. The DPA results are consistent with the findings of the ss-DFT simulations that revealed the DPA molecule to have relatively less conformational strain in the MPA:DPA cocrystal. These results demonstrate that MPA conformational flexibility is a property of critical importance for understanding the stability of the cocrystal.

**Figure 7:**
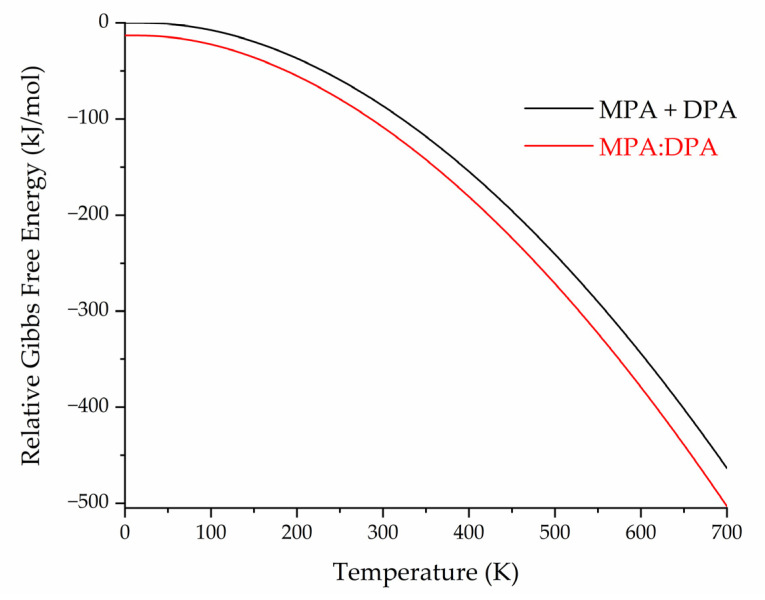
Relative Gibbs free energy curves for the linear combination of pure MPA and pure DPA (black) and the MPA:DPA cocrystal (red) as a function of temperature. Energies are per MPA and DPA pair.

Collectively, the electronic (cohesive and conformational) energies of the solids can be combined with the results of the vibrational analyses to produce Gibbs free energy values as a function of temperature. This provides a more holistic representation of crystal stability and can more easily be related to experimental observations. Figure 7 shows the calculated Gibbs free energy data relevant to the MPA:DPA cocrystal in two ways. One data series is a simple linear combination of the separately calculated pure MPA and pure DPA Gibbs free energies (MPA + DPA). The second series is the calculated Gibbs free energy of the true MPA:DPA cocrystal. The MPA:DPA Gibbs free energy is clearly lower than the MPA + DPA separate components over the entire temperature range, indicating that MPA:DPA cocrystallization is thermodynamically preferred versus the formation of pure crystals. At 0 K, the MPA:DPA cocrystal is 13.17 kJ/mol lower in energy (per MPA and DPA pair) than the MPA + DPA combination of the pure species, and the free energy difference increases to 21.87 kJ/mol at 295 K.

The conformational and cohesive energy results provide physical insights concerning the important structural changes in the crystallization process of MPA:DPA, but it is not clear that the knowledge is directly applicable to understanding the aqueous solubilities of related compounds such as MMF (C_23_H_31_NO_7_, CAS #: 128794-94-5). While not the focus of the present work, it is worthwhile to briefly consider these results in the context of their relevance to the MMF molecule, as it is the commonly prescribed prodrug form of MPA. The same experimental and computational methods applied to the MPA and DPA systems have been applied to MMF. Experimental and simulated Raman spectra of MMF are provided in the Supplementary Materials, and only the ss-DFT energies of the pure MMF crystal will be considered here [34]. Since the molecules have different chemical formulas, the cohesive energies per atom of the various solids are the most straightforward to compare. As a simple first-order approximation, the cohesive energies are the calculated property most closely related to the solubilities of the compounds. However, the complete solvation process is far more complicated to model accurately [35]. The dissolution profiles of MMF, MPA:DPA, and MPA have been previously reported with their aqueous solubilities being in that order, from low to high [15]. The relative cohesive energies of MPA and MPA:DPA do follow the experimental solubility trend, supporting the basic idea that greater intermolecular forces within the MPA:DPA solid could make it more resistant to dissolution. With a relative cohesive energy per atom of −3.73 kJ/mol, the relatively weak solid-state cohesion of MMF suggests that it should have high solubility, but this is not found experimentally. The reduction of cohesive energy in the MMF solid is related to the decrease in hydrogen bonding interactions in its crystal structure compared to both MPA and MPA:DPA, as evident from its molecular structure (see Figure 1). With this limitation, there are fewer attractive solvation interactions between MMF and water, thereby leading to its very low aqueous solubility. Ultimately, these results demonstrate that while structures and energies of the same compounds can be successfully compared in different solid-state arrangements and environments, it is not always possible to directly translate this knowledge to other systems with distinctly different chemical characters regardless of their pharmaceutical relevance.

## 4. Conclusions

The LFRS measurement of lattice vibrations in pure and cocrystallized pharmaceutical solids provides unique spectral fingerprints that can be used in analytical applications for detection and identification of complex materials. The sub-200 cm^−1^ peak positions and intensities in the Raman spectra of pure MPA, pure DPA, and cocrystalline MPA:DPA are distinctly different from each other and indicative of the various large-amplitude motions within the solids. The LFRS data also serve as definitive benchmarks for judging the quality of quantum mechanical simulations of the pure and cocrystal structures and the intermolecular forces that exist between crystal components. The successful assignment of these complicated spectra to specific atomic motions would not be possible without a modeling approach that offers an accurate and reliable representation of the crystalline environment. The careful use of ss-DFT simulations yielded very good reproductions of the crystal structures and Raman spectra of all the solids studied here, revealing that the observed vibrations in this region are primarily internal torsions and hindered rotations of the molecules. The high correlation of the experiments and simulations supported the further exploration of the energetic origins of the MPA:DPA cocrystal.

The conformational and cohesive energies that were found in the solids considered here follow a pattern that is not unusual in the general formation of molecular crystals and cocrystals. This pattern is one in which the molecular components sacrifice conformational energy to achieve enhanced intermolecular interactions and leverage these energetic benefits to create a stable solid arrangement. However, there is a balance between the two factors. The placement of the hydrogen-bonding carboxylic acid group at the terminus of the conformationally flexible hydrocarbon chain of MPA is the combined core feature that promotes the formation of the MPA:DPA cocrystal. Now that this balance has been quantified, the concept can readily be used in the rational design of future MPA cocrystals as well as generally applied to other pharmaceutical solids.

## Figures and Tables

**Table 1:** Comparison of the experimental and ss-DFT optimized crystallographic unit cell dimensions (Å), angles (°), and volumes (V, Å^3^) of MPA, DPA, and MPA:DPA.

	MPA			DPA			MPA:DPA		
	Exp. [15]	ss-DFT	% Error	Exp. [32]	ss-DFT	% Error	Exp. [15]	ss-DFT	% Error
*a*	7.3469	7.15191	−2.7	18.416	18.17738	−1.3	11.6989	11.55358	−1.2
*b*	9.5469	9.52802	−0.2	12.294	12.15401	−1.1	14.8760	15.04209	1.1
*c*	11.6228	11.49439	−1.1	7.691	7.56011	−1.7	15.019	14.78744	−1.5
*α*	102.809	101.8443	−0.9	90	90	-	90	90	-
*β*	90.902	92.3840	1.6	90	90	-	107.925	108.2683	0.3
*γ*	90.873	88.8070	−2.3	90	90	-	90	90	-
*V*	794.69	765.87	−3.6	1741.29	1670.24	−4.1	2486.93	2440.38	−1.9

**Table 2:** RMSD analyses of the differences in bond lengths, bond angles, and torsional angles between experimental MPA [15], DPA [32], and MPA:DPA [15] and ss-DFT optimized crystal structures.

	MPA	DPA	MPA:DPA
Bond lengths (Å)	0.023	0.018	0.018
Bond angles (°)	0.650	0.619	0.477
Torsional angles (°)	4.916	0.972	3.864

**Table 3:** Comparison of intermolecular hydrogen bond donor (D) to acceptor (A) distances and angles (D-H···A geometry) for experimental MPA [15], DPA [32], and MPA:DPA [15] and ss-DFT optimized crystal structures.

	Atoms	D-H (Å)		H···A (Å)		D-H···A (^o^)		D···A (Å)	
		Exp.	ss-DFT	Exp.	ss-DFT	Exp.	ss-DFT	Exp.	ss-DFT
MPA	O4-H1···O6	0.82	0.9880	2.443	2.1901	127.7	130.11	3.015	2.9257
	O5-H2···O6	0.82	1.0211	1.902	1.6300	175.3	176.14	2.720	2.6500
DPA	N2-H3···N1	0.87	1.0482	2.180	1.8634	172.4	172.26	3.043	2.9055
MPA:DPA	O4-H1···O5	0.82	0.9862	2.422	2.2969	121.2	117.37	2.933	2.8864
	O5-H2···N1	0.95	1.0915	1.700	1.5061	172.9	171.33	2.642	2.5903
	N2-H3···O6	0.85	1.0384	2.026	1.8196	179.6	179.18	2.879	2.8579

**Table 4:** Correlation of experimental and ss-DFT simulated Raman-active vibrational frequencies (cm^−1^) for pure crystals of MPA and DPA with mode descriptions. Crystallographic axes are indicated as needed.

	Exp. freq	ss-DFT freq	Mode Description
MPA	33.6	35.80	ring-chain torsion
	40.1	33.13	rotation about *a*
	53.3	51.88	rotation about *c*
	63.8	61.89 65.36	ring-chain torsion ring-chain torsion
	77.4	74.83	ring-chain torsion
	85.2	87.91	ring-chain torsion
DPA	26.4	26.41 28.12	rotation about *b* translation along *b*
	36.0	36.54	translation along *a*
	47.9	48.78	rotation about *c*
	52.7	53.37	rotation about *b*
	58.5	60.17	rotation about *b*
	70.7	72.43 75.52	rotation about *c* rotation about *c*
	73.7	77.83	ring torsion at amine linkage
	81.5	82.89	rotation about *c*
	87.6	91.95	ring torsion at amine linkage
	100–115 (broad)	105.10 108.18 112.71 113.37	ring torsion at amine linkage ring torsion at amine linkage ring torsion at amine linkage rotation about *b*
	128.8	132.30	bending at amine linkage

**Table 5:** Correlation of experimental and ss-DFT simulated Raman-active vibrational frequencies (cm^−1^) for MPA:DPA with mode descriptions. Crystallographic axes and molecular localization of vibrations are indicated as needed.

Exp. freq	ss-DFT freq	Mode Description
32.7	32.20 37.09 39.00	rotation about *b* rotation about *c* rotation about *b*
45.3	45.26	ring-chain torsion (MPA)
49.2	50.29 50.60	rotation about *b* translation along *b*
57.2	59.07	rotation about *a*
75–85 (broad)	76.64 79.24 79.99	methoxy torsion (MPA) methoxy torsion (MPA) intra-chain torsion (MPA)
95–105 (broad)	94.30 98.97 101.86	ring-ring torsion (DPA) ring-ring torsion (DPA) ring-ring bending (DPA)
122.1	118.83	ring-ring torsion (DPA)

## Data Availability

The data presented in this study are available within the article and in the Supplementary Materials file. Any additional information can be obtained upon request.

## Supplementary Materials

The following supporting information can be downloaded at: https://www.mdpi.com/article/10.3390/pharmaceutics15071924/s1, PXRD patterns for MPA, DPA, MPA:DPA, and MMF. Crystallographic unit cell images and lattice parameter information for MPA, DPA, MPA:DPA, and MMF. Fractional atomic coordinates for MPA, DPA, MPA:DPA, and MMF. Experimental and simulated Raman spectra of MMF. Room-temperature Raman spectra of MPA, DPA, and MPA:DPA. Lists of all calculated vibrational frequencies and intensities (IR and Raman) for MPA, DPA, MPA:DPA, and MMF with mode symmetries.
