# Supplementary material for: Low-Frequency Raman Spectroscopy of Pure and Cocrystallized Mycophenolic Acid
Source: Pharmaceutics. 2023 Jul 11;15(7):1924. doi: 10.3390/pharmaceutics15071924 (PMC10384077; doi:10.3390/pharmaceutics15071924)
Supplement: Supplementary file 1 [file pharmaceutics-15-01924-s001.zip › pharmaceutics-2440231-supplementary.pdf]

Low-Frequency Raman Spectroscopy of Pure and Cocrystallized Mycophenolic Acid

Catherine S. Wallace, Margaret P. Davis, and Timothy M. Korter

Department of Chemistry, Syracuse University, 1-133 Center for Science and Technology, Syracuse, New York 13244-4100, United States

Supplementary Materials

Figure S1. PXRD patterns of MPA sample (red) and its published CSD structure[SR1] (black).

Figure S2. PXRD patterns of DPA sample (red) and its published CSD structure[SR2] (black).

Figure S3. PXRD patterns of MPA:DPA sample (red) and its published CSD structure[SR1] (black).

Figure S4. PXRD patterns of MMF sample (red) and its published CSD structure[SR3] (black).

Figure S5. Crystallographic unit cell of MPA based on ss-DFT optimized structure looking down the *a*-axis with *b* (green) and *c* (blue). Atom colors: red = O, gray = C, white = H.

Figure S6. Crystallographic unit cell of DPA based on ss-DFT optimized structure looking down the *b*-axis with *a* (red) and *c* (blue). Atom colors: purple = N, gray = C, white = H.

Figure S7. Crystallographic unit cell of MPA:DPA based on ss-DFT optimized structure with *a* (red), *b* (green), and *c* (blue) axes. Atom colors: red = O, purple = N, gray = C, white = H.

Figure S8. Crystallographic unit cell of MMF based on ss-DFT optimized structure with *a* (red), *b* (green), and *c* (blue) axes. Atom colors: red = O, purple = N, gray = C, white = H.

Figure S9. Comparison of experimental (78 K, black) and simulated (red) Raman spectra of MMF. Mycophenolate mofetil (98%) was purchased through Fisher Scientific and used without further purification. An empirical Lorentzian line shape with a full-width-at-half-maximum of 2 cm^{-1} was added for comparison to experiment. Spectral intensities have been normalized to 1.0 within each data set. No corrections have been made for baseline offset or interference from atmospheric gases.

Table S1. Lattice parameters including space group, unit cell dimensions (Å), angles (°), volume (V, $Å^3$), and density (g/cm^3) of ss-DFT optimized MPA, DPA. MPA:DPA, and MMF.

Table S2. Fractional atomic coordinates for the asymmetric unit of ss-DFT optimized MPA.

Table S3. Fractional atomic coordinates for the asymmetric unit of ss-DFT optimized DPA.

Table S4. Fractional atomic coordinates for the asymmetric unit of ss-DFT optimized MPA:DPA.

Table S5. Fractional atomic coordinates for the asymmetric unit of ss-DFT optimized MMF.

Table S6. Solid-state DFT IR-active modes with frequencies (cm^{-1}) and infrared absorption intensities (km/mol) for MPA.

Table S7. Solid-state DFT Raman-active modes with frequencies (cm^{-1}) and relative Raman scattering intensities (normalized to 1000) for MPA at 78 K.

Table S8. Solid-state DFT IR-active modes with frequencies (cm^{-1}) and infrared absorption intensities (km/mol) for DPA.

Table S9. Solid-state DFT Raman-active modes with frequencies (cm^{-1}) and relative Raman scattering intensities (normalized to 1000) for DPA at 78 K.

Table S10. Solid-state DFT IR-active modes with frequencies (cm^{-1}) and infrared absorption intensities (km/mol) for MPA:DPA.

Table S11. Solid-state DFT Raman-active modes with frequencies (cm^{-1}) and relative Raman scattering intensities (normalized to 1000) for MPA:DPA at 78 K.

Table S12. Solid-state DFT IR-active modes with frequencies (cm^{-1}) and infrared absorption intensities (km/mol) for MMF.

Table S13. Solid-state DFT Raman-active modes with frequencies (cm^{-1}) and relative Raman scattering intensities (normalized to 1000) for MMF at 78 K.

Supplementary Materials References

SR1. Zeng, Q.-Z.; Ouyang, J.; Zhang, S.; Zhang, L., Structural characterization and dissolution profile of mycophenolic acid cocrystals. *European Journal of Pharmaceutical Sciences* **2017,** *102*, 140-146.

SR2. Johnson, J. E.; Jacobson, R. A., The crystal and molecular structure of di(2-pyridyl)amine. *Acta Crystallographica Section B* **1973,** *29* (8), 1669-1674.

SR3. Yathirajan, H. S.; Nagaraj, B.; Gaonkar, S. L.; Narasegowda, R. S.; Nagaraja, P.; Bolte, M., Mycophenolate mofetil. *Acta Crystallographica Section E* **2004,** *60* (12), o2223-o2224.

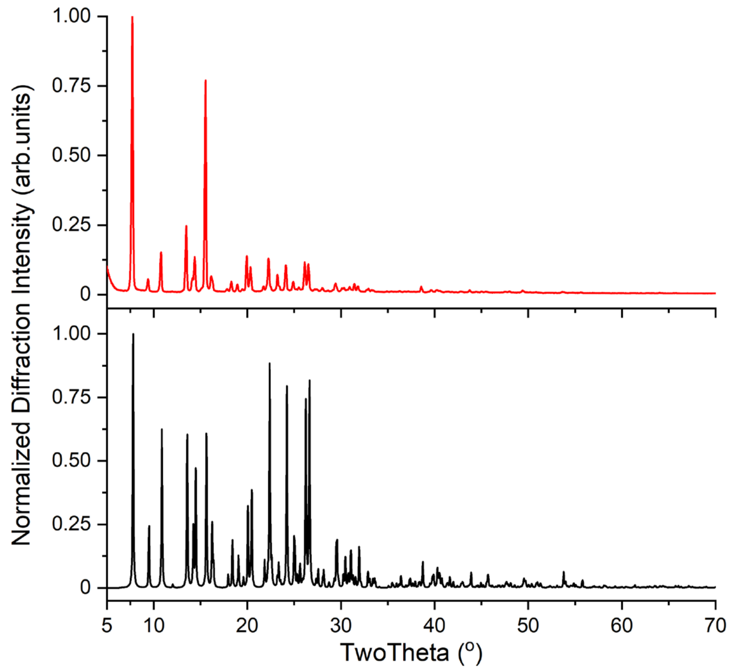

Figure S1. PXRD patterns of MPA sample (red) and its published CSD structure[SR1] (black).

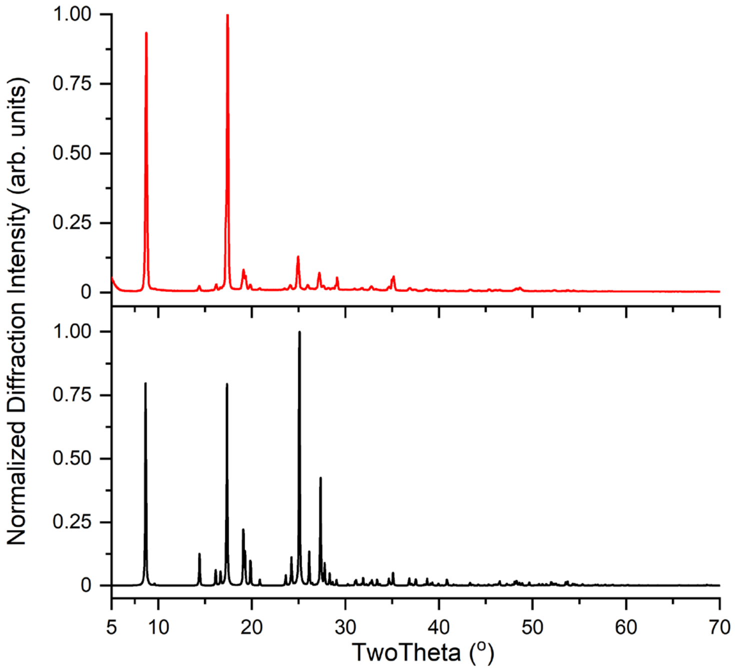

Figure S2. PXRD patterns of DPA sample (red) and its published CSD structure[SR2] (black).

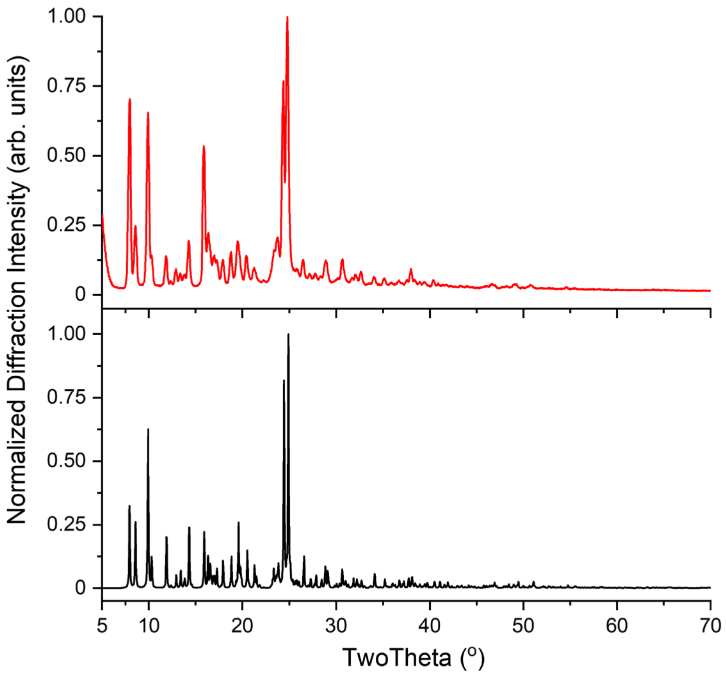

Figure S3. PXRD patterns of MPA:DPA sample (red) and its published CSD structure[SR1] (black).

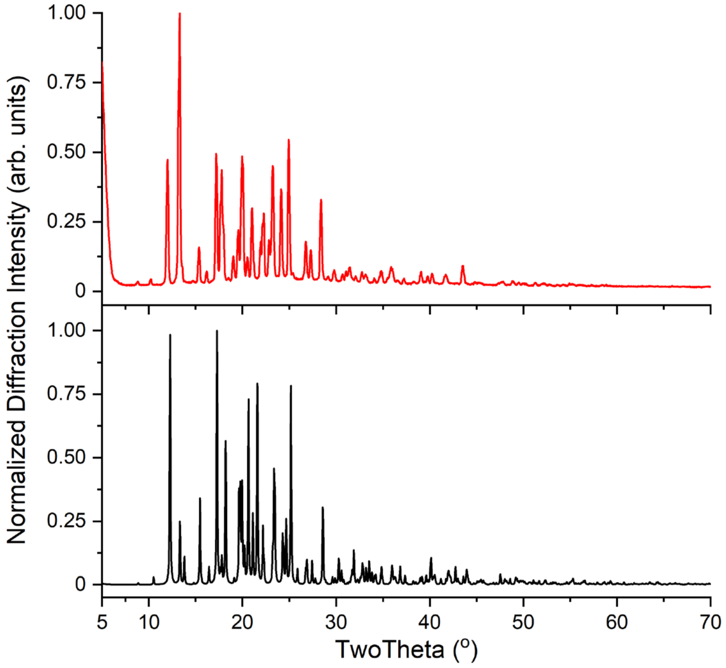

Figure S4. PXRD patterns of MMF sample (red) and its published CSD structure[SR3] (black).

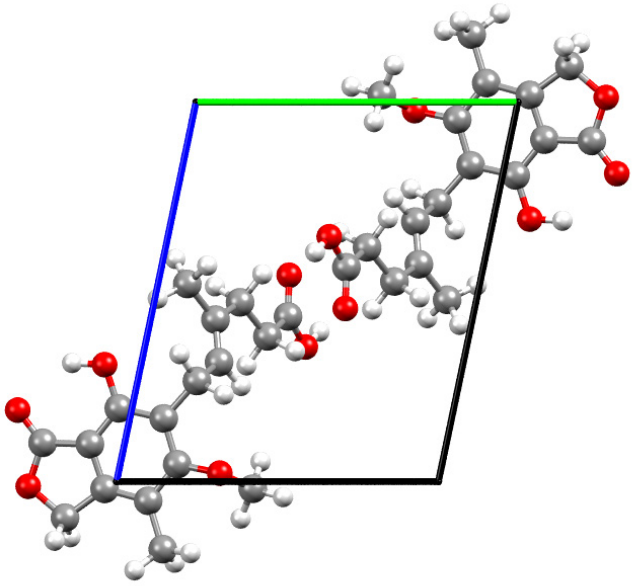

Figure S5. Crystallographic unit cell of MPA based on ss-DFT optimized structure looking down the *a*-axis with *b* (green) and *c* (blue). Atom colors: red = O, gray = C, white = H.

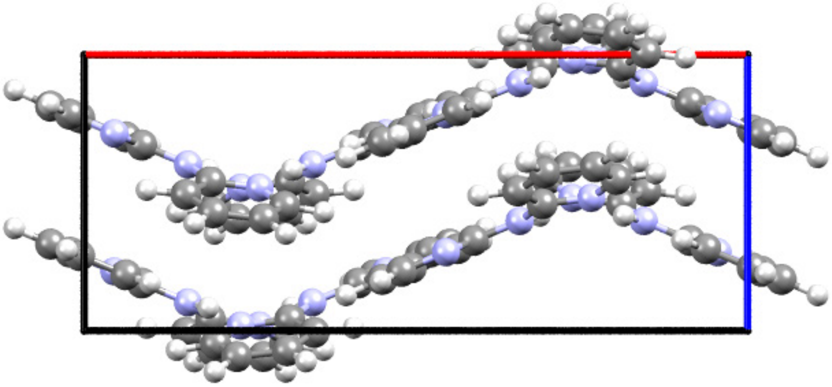

Figure S6. Crystallographic unit cell of DPA based on ss-DFT optimized structure looking down the *b*-axis with *a* (red) and *c* (blue). Atom colors: purple = N, gray = C, white = H.

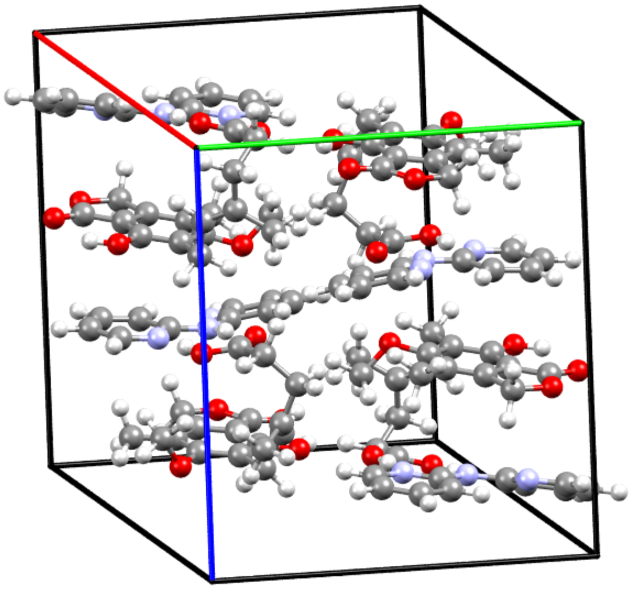

Figure S7. Crystallographic unit cell of MPA:DPA based on ss-DFT optimized structure with *a* (red), *b* (green), and *c* (blue) axes. Atom colors: red = O, purple = N, gray = C, white = H.

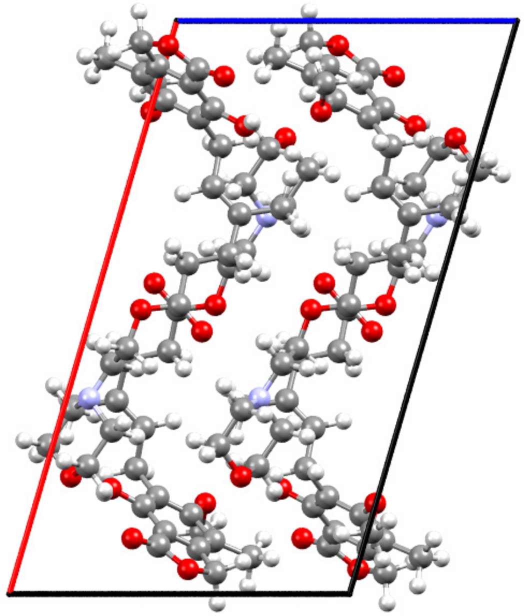

Figure S8. Crystallographic unit cell of MMF based on ss-DFT optimized structure with *a* (red), *b* (green), and *c* (blue) axes. Atom colors: red = O, purple = N, gray = C, white = H.

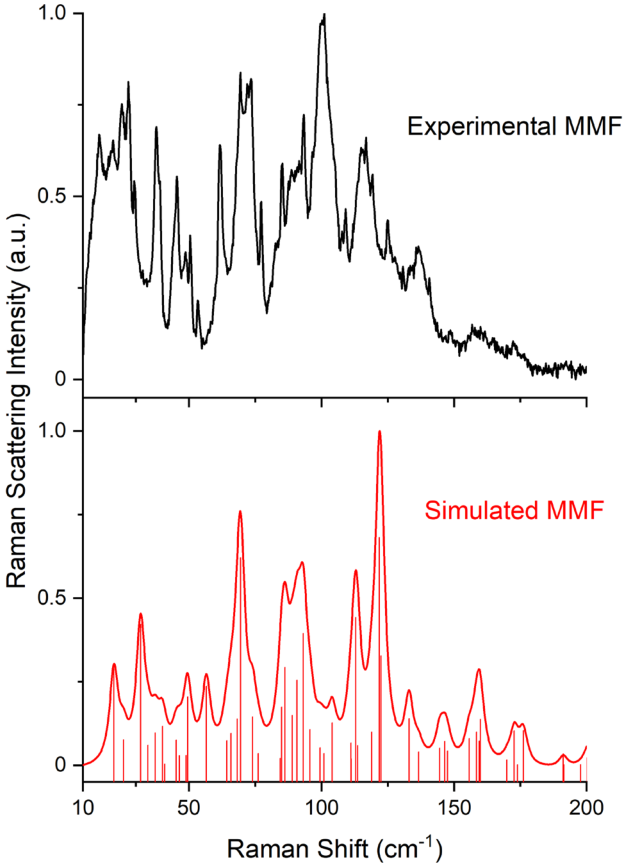

Figure S9. Comparison of experimental (78 K, black) and simulated (red) Raman spectra of MMF. Mycophenolate mofetil (98%) was purchased through Fisher Scientific and used without further purification. An empirical Lorentzian line shape with a full-width-at-half-maximum of 2 cm^{-1} was added for comparison to experiment. Spectral intensities have been normalized to 1.0 within each data set. No corrections have been made for baseline offset or interference from atmospheric gases.

Table S1. Lattice parameters including space group, unit cell dimensions (Å), angles (°), volume (V, Å^3), and density (g/cm^3) of ss-DFT optimized MPA, DPA. MPA:DPA, and MMF.

	MPA	DPA	MPA:DPA	MMF
Space group	$P\bar{1}$	$Pccn$	$P2_1/c$	$P2_1/c$
a	7.15191	18.17738	11.55358	20.85478
b	9.52802	12.15401	15.04209	9.35033
c	11.49439	7.56011	14.78744	11.87535
α	101.8443	90	90	90
β	92.3840	90	108.2683	106.2815
γ	88.8070	90	90	90
Volume	765.87	1670.24	2440.38	2222.82
Density	1.39	1.36	1.34	1.30

Table S2. Fractional atomic coordinates for the asymmetric unit of ss-DFT optimized MPA.

Atom (atomic number)	X/A	Y/B	Z/C
8	3.42347E-01	-3.53910E-01	-1.88291E-01
8	3.64534E-01	-2.71458E-01	1.24613E-02
8	9.61474E-02	3.15133E-01	-2.22210E-02
8	2.08669E-01	-1.13255E-01	-3.08077E-01
8	-9.40387E-02	4.98594E-01	-3.54723E-01
8	-1.86854E-01	4.01908E-01	4.56171E-01
6	3.25210E-01	-2.55108E-01	-1.02220E-01
6	3.31985E-01	-1.36745E-01	9.52263E-02
6	2.69990E-01	-3.26312E-02	1.92692E-02
6	2.17396E-01	1.10664E-01	5.06881E-02
6	1.64505E-01	1.76987E-01	-4.56167E-02
6	1.63493E-01	1.05325E-01	-1.66469E-01
6	2.13974E-01	-4.10235E-02	-1.93108E-01
6	2.67590E-01	-1.07212E-01	-9.86810E-02
6	1.11648E-01	1.84114E-01	-2.65207E-01
6	2.80316E-01	2.40723E-01	-3.14799E-01
6	3.37159E-01	2.01894E-01	-4.27032E-01
6	-4.97042E-01	2.67007E-01	-4.72445E-01
6	-3.81683E-01	3.74121E-01	-3.82614E-01
6	-2.12418E-01	4.25372E-01	-4.35227E-01
6	2.08721E-01	1.87242E-01	1.78118E-01
6	2.31086E-01	4.27545E-01	1.89345E-02
6	2.38084E-01	9.11920E-02	4.79206E-01
1	4.63658E-01	-1.07144E-01	1.46512E-01
1	2.24020E-01	-1.54744E-01	1.55896E-01
1	3.12436E-02	1.12611E-01	-3.35949E-01
1	2.09278E-02	2.76107E-01	-2.28884E-01
1	3.56003E-01	3.24218E-01	-2.52057E-01
1	-4.04946E-01	1.78617E-01	4.85327E-01
1	4.53560E-01	3.17869E-01	4.53528E-01
1	-3.31799E-01	3.28228E-01	-3.06264E-01
1	-4.65623E-01	4.69571E-01	-3.43662E-01
1	1.68630E-01	1.13047E-01	2.33919E-01
1	1.06395E-01	2.75771E-01	1.88563E-01
1	3.44449E-01	2.33182E-01	2.13784E-01
1	3.13098E-01	4.09350E-01	9.74934E-02
1	1.49748E-01	-4.72853E-01	4.09751E-02
1	3.25934E-01	4.34410E-01	-5.22666E-02
1	1.73116E-01	8.79697E-03	-4.82336E-01

1	3.37148E-01	3.73077E-02	4.13110E-01
1	1.27473E-01	1.40892E-01	4.30829E-01
1	2.40293E-01	-2.15601E-01	-3.12512E-01
1	1.58275E-02	-4.67336E-01	-3.95267E-01

Table S3. Fractional atomic coordinates for the asymmetric unit of ss-DFT optimized DPA.

Atom (atomic number)	X/A	Y/B	Z/C
7	-4.54700E-01	-1.17486E-01	2.84636E-01
7	-3.43656E-01	-1.91381E-01	3.97591E-01
7	-2.35985E-01	-1.08008E-01	4.81366E-01
6	4.78427E-01	-1.36822E-01	2.15051E-01
6	-1.98073E-01	-2.29638E-02	-4.51824E-01
6	4.49209E-01	-2.40910E-01	1.85945E-01
6	4.93152E-01	-3.32069E-01	2.27878E-01
6	-4.37415E-01	-3.14559E-01	2.97308E-01
6	-4.13150E-01	-2.05514E-01	3.27122E-01
6	-3.09640E-01	-9.73610E-02	4.59822E-01
6	-3.46755E-01	3.49982E-04	-4.93684E-01
6	-3.05969E-01	8.75538E-02	-4.27003E-01
6	-2.29889E-01	7.72251E-02	-4.06274E-01
1	4.46641E-01	-6.35978E-02	1.80115E-01
1	-1.39305E-01	-3.69077E-02	-4.29833E-01
1	3.94621E-01	-2.50375E-01	1.28400E-01
1	4.73164E-01	-4.15895E-01	2.07318E-01
1	-4.00886E-01	-3.82957E-01	3.27898E-01
1	-3.12998E-01	-2.63446E-01	4.16764E-01
1	-4.05994E-01	5.12190E-03	4.89879E-01
1	-3.34249E-01	1.63018E-01	-3.88553E-01
1	-1.96257E-01	1.42943E-01	-3.51021E-01

Table S4. Fractional atomic coordinates for the asymmetric unit of ss-DFT optimized MPA:DPA.

Atom (atomic number)	X/A	Y/B	Z/C
8	-1.69151E-01	-3.76711E-02	3.80493E-01
8	-4.70331E-02	8.17911E-02	3.82161E-01
8	-4.33311E-01	3.40322E-01	3.95376E-01
8	-4.17136E-01	2.50890E-02	3.78057E-01
8	4.18619E-01	3.35045E-01	9.53359E-02
8	4.33640E-01	1.88419E-01	6.87900E-02
6	-1.55798E-01	4.32568E-02	3.79871E-01
6	-5.98304E-02	1.78124E-01	3.79253E-01
6	-1.87582E-01	1.95885E-01	3.79354E-01
6	-2.47476E-01	2.75186E-01	3.83484E-01
6	-3.70056E-01	2.66192E-01	3.82999E-01
6	-4.29058E-01	1.83311E-01	3.79280E-01
6	-3.62994E-01	1.05247E-01	3.78195E-01
6	-2.42889E-01	1.13224E-01	3.77524E-01
6	4.38700E-01	1.76925E-01	3.74185E-01
6	3.60963E-01	1.57401E-01	2.73519E-01
6	2.59063E-01	1.99990E-01	2.22514E-01
6	2.02656E-01	1.78288E-01	1.18068E-01
6	2.35783E-01	2.49330E-01	5.49270E-02
6	3.72307E-01	2.54694E-01	7.40591E-02
6	-1.84205E-01	3.63640E-01	3.92477E-01
6	-4.73476E-01	4.00861E-01	3.16250E-01
6	1.98225E-01	2.72801E-01	2.60341E-01
1	-4.40027E-02	2.00994E-01	3.13337E-01
1	1.04361E-02	2.05097E-01	4.41745E-01
1	4.29298E-01	1.23780E-01	4.22753E-01
1	4.12136E-01	2.39378E-01	4.00177E-01
1	3.95714E-01	1.04812E-01	2.37827E-01
1	1.02493E-01	1.74857E-01	9.90479E-02
1	2.34897E-01	1.13167E-01	1.02108E-01
1	1.95376E-01	2.31030E-01	-2.06703E-02
1	2.01116E-01	3.15229E-01	6.53367E-02
1	-8.84422E-02	3.57752E-01	4.37117E-01
1	-2.27525E-01	4.12985E-01	4.26051E-01
1	-1.84723E-01	3.89890E-01	3.22740E-01
1	-4.01284E-01	4.14432E-01	2.84367E-01
1	-4.98336E-01	4.62764E-01	3.44607E-01
1	4.46731E-01	3.73564E-01	2.61454E-01

1	2.47403E-01	2.89210E-01	3.34677E-01
1	1.05049E-01	2.52361E-01	2.56259E-01
1	1.89100E-01	3.34365E-01	2.18376E-01
1	-4.83515E-01	3.36239E-01	1.06108E-01
1	-3.57591E-01	-2.32266E-02	3.83675E-01
7	-3.50513E-01	3.52936E-01	1.20048E-01
7	-3.07520E-01	2.04098E-01	1.17479E-01
7	-1.24433E-01	1.21538E-01	1.50034E-01
6	-3.19803E-01	4.39520E-01	1.23335E-01
6	-2.03005E-01	4.68504E-01	1.31116E-01
6	-1.14034E-01	4.03921E-01	1.35636E-01
6	-1.43732E-01	3.14375E-01	1.33111E-01
6	-2.64593E-01	2.89952E-01	1.24635E-01
6	-2.46918E-01	1.24471E-01	1.17841E-01
6	-3.19366E-01	4.83602E-02	8.40341E-02
6	-2.62001E-01	-3.22813E-02	8.32764E-02
6	-1.34238E-01	-3.60146E-02	1.16806E-01
6	-7.08056E-02	4.22197E-02	1.49131E-01
1	-3.94139E-01	4.85691E-01	1.19096E-01
1	-1.82110E-01	-4.60801E-01	1.32942E-01
1	-2.14403E-02	4.23574E-01	1.40168E-01
1	-7.76660E-02	2.62130E-01	1.36521E-01
1	-4.18203E-01	5.51725E-02	5.70461E-02
1	-3.16712E-01	-9.10407E-02	5.45701E-02
1	-8.50267E-02	-9.81137E-02	1.18525E-01
1	2.87944E-02	4.22213E-02	1.76621E-01
1	-4.01649E-01	1.98645E-01	9.91836E-02

Table S5. Fractional atomic coordinates for the asymmetric unit of ss-DFT optimized MMF.

Atom (atomic number)	X/A	Y/B	Z/C
8	-4.31013E-02	2.21395E-01	-4.95875E-01
8	-9.68053E-02	1.76389E-01	3.13422E-01
8	-1.83661E-01	4.44003E-01	2.08680E-01
8	-1.58008E-01	-1.94357E-01	4.98093E-01
8	4.95943E-01	-4.69142E-01	3.57317E-01
8	4.58000E-01	-4.39232E-01	1.59309E-01
8	2.04661E-01	-2.97259E-01	4.19894E-01
7	3.41191E-01	-3.64098E-01	4.32972E-01
6	-8.46028E-02	2.59593E-01	3.96687E-01
6	-1.07640E-01	4.05167E-01	4.03528E-01
6	-7.91031E-02	4.56636E-01	-4.82777E-01
6	-3.70432E-02	3.39532E-01	-4.13785E-01
6	-9.28720E-02	-4.08812E-01	-4.46400E-01
6	-1.38222E-01	-3.25657E-01	4.67029E-01
6	-1.68545E-01	-3.74187E-01	3.51767E-01
6	-1.53371E-01	4.87556E-01	3.19026E-01
6	-6.46328E-02	-3.59943E-01	-3.21843E-01
6	-2.18679E-01	-2.83344E-01	2.64508E-01
6	-2.89776E-01	-3.29619E-01	2.50755E-01
6	-3.37653E-01	-3.49454E-01	1.49111E-01
6	-3.26614E-01	-3.26215E-01	3.03287E-02
6	-4.08249E-01	-3.93274E-01	1.43647E-01
6	-4.23862E-01	-4.06849E-01	2.60881E-01
6	-4.96231E-01	-4.39865E-01	2.49736E-01
6	-1.08849E-01	-8.16988E-02	-4.88615E-01
6	4.28436E-01	-4.89574E-01	3.65768E-01
6	4.01608E-01	-3.46903E-01	3.95332E-01
6	2.85273E-01	-4.19704E-01	3.38843E-01
6	2.23095E-01	-4.32756E-01	3.80695E-01
6	2.59180E-01	-2.43288E-01	-4.86566E-01
6	3.22176E-01	-2.26705E-01	4.74385E-01
1	-1.76039E-01	3.41740E-01	1.92471E-01
1	1.62664E-02	3.66985E-01	-3.81054E-01
1	-5.57959E-02	3.04056E-01	-3.40589E-01
1	-6.17602E-02	-4.49354E-01	-2.60485E-01
1	-9.56448E-02	-2.75648E-01	-2.99780E-01
1	-1.38827E-02	-3.16631E-01	-3.06464E-01
1	-2.07335E-01	-2.85991E-01	1.79562E-01
1	-2.12330E-01	-1.72393E-01	2.96701E-01

1	-3.01800E-01	-3.46476E-01	3.33652E-01
1	-3.40579E-01	-2.15859E-01	2.16299E-04
1	-3.58523E-01	-3.98219E-01	-3.52143E-02
1	-2.74478E-01	-3.42175E-01	3.02185E-02
1	-4.19736E-01	-4.94688E-01	9.45201E-02
1	-4.43241E-01	-3.16023E-01	8.80955E-02
1	-3.92905E-01	-4.88403E-01	3.18235E-01
1	-4.12543E-01	-3.06031E-01	3.10836E-01
1	-5.97318E-02	-1.15099E-01	-4.32630E-01
1	-1.27649E-01	7.74047E-03	-4.47645E-01
1	-1.03682E-01	-4.94551E-02	4.25317E-01
1	4.33078E-01	4.35306E-01	4.38397E-01
1	3.97670E-01	4.64296E-01	2.83331E-01
1	4.39994E-01	-2.98026E-01	4.68295E-01
1	3.93386E-01	-2.73662E-01	3.18485E-01
1	2.97931E-01	4.73699E-01	3.12251E-01
1	2.74407E-01	-3.48835E-01	2.60353E-01
1	1.80211E-01	-4.66967E-01	3.09195E-01
1	2.31645E-01	4.89822E-01	4.53603E-01
1	2.67819E-01	-3.17287E-01	-4.11100E-01
1	2.42976E-01	-1.39159E-01	-4.61589E-01
1	3.12854E-01	-1.44417E-01	4.03990E-01
1	3.63328E-01	-1.88355E-01	-4.51887E-01

Table S6. Solid-state DFT IR-active modes with frequencies (cm^{-1}) and infrared absorption intensities (km/mol) for MPA.

Frequency	IR Intensity
30.90	1.34
44.38	1.00
62.72	2.20
73.67	9.51
75.98	7.21
92.61	5.38
99.67	2.40
105.25	17.67
115.57	24.08
125.26	2.19
133.37	9.74
156.90	8.66
159.44	5.77
166.62	5.57
176.23	2.42
183.32	10.53
186.33	6.75
198.00	17.45
200.42	16.82
228.70	30.64
241.11	47.95
247.80	29.34
254.27	35.12
274.07	15.61
290.13	11.12
343.94	21.33
358.61	51.45
365.13	12.74
370.53	19.96
393.64	18.80
434.08	13.46
442.34	42.11
470.40	41.21
513.71	12.21
528.26	47.09
544.64	28.91
561.87	16.31
567.43	7.48
570.80	1.62
635.85	5.65
649.74	37.72
674.48	20.72
684.77	21.60
712.13	326.86
726.06	44.93
737.12	92.40
738.42	26.15
744.45	16.47
801.34	18.71
821.59	130.95
856.53	114.33
904.41	31.75
915.43	14.21
943.97	142.60
954.39	221.33
964.63	10.08
967.40	11.32
982.91	27.53
988.84	4.78
1003.04	492.83
1010.29	469.34
1022.99	61.90
1039.50	6.06
1048.52	262.34
1059.62	390.62
1082.72	71.98
1092.02	279.16
1125.74	282.41
1133.78	61.14
1156.56	192.38
1164.72	204.32
1170.65	16.74
1181.43	182.96
1197.18	598.22
1222.64	155.34
1239.72	287.89
1265.87	62.15
1278.20	57.53
1293.83	49.54
1307.53	348.90
1315.08	519.53
1318.50	167.25
1330.66	180.01
1359.35	97.27
1370.30	69.60
1375.25	61.62
1376.22	31.28
1390.01	280.02
1404.57	179.39
1421.32	87.58
1426.59	208.35
1427.04	145.96
1432.63	14.59
1433.73	60.34
1436.96	17.84
1443.12	86.29
1449.32	95.31
1454.57	30.69
1458.21	190.65
1462.74	5.26
1476.92	63.41
1599.48	383.56
1613.54	303.61
1666.21	1656.96
1677.97	179.40
1715.05	1231.25
2893.89	5614.39
2951.82	45.58
2959.65	152.44
2967.39	50.11
2978.68	30.39
2984.07	23.08
2986.32	59.61
2988.29	100.75
2993.78	6.38
3012.89	27.72

3016.12	50.13
3028.04	8.88
3041.27	2.25
3046.12	32.75

3066.51	18.14
3068.26	39.99
3085.25	3.22
3088.91	23.12

3121.74	1.19
3449.84	927.10

Table S7. Solid-state DFT Raman-active modes with frequencies (cm^{-1}) and relative Raman scattering intensities (normalized to 1000) for MPA at 78 K.

Frequency	Raman Intensity
33.13	1000.00
35.80	429.41
51.88	91.81
61.89	504.13
65.36	377.24
74.83	115.93
87.91	179.86
97.45	231.76
107.44	117.71
113.24	114.70
122.11	100.22
122.69	210.57
141.43	55.18
148.16	69.83
154.29	69.70
159.01	31.76
173.86	76.19
178.20	39.81
184.55	106.57
194.38	18.91
200.06	44.33
207.12	32.27
231.67	117.89
238.34	25.12
246.92	47.33
263.00	115.91
288.28	10.29
297.43	55.93
342.92	42.40
351.49	35.80
363.56	114.44
370.02	70.60
392.63	117.82
430.57	28.65
444.58	46.57
470.17	152.34
503.77	15.31
516.43	54.70
545.63	258.27
561.53	143.63
570.06	106.29
573.24	24.29
634.63	55.47
644.38	18.66
659.10	56.40
682.56	30.89
711.76	81.52
729.96	195.26
734.72	43.89
740.80	39.28
746.92	9.63
801.30	44.96
821.30	19.90
857.69	120.27
903.02	103.15
910.29	87.50
944.87	31.54
953.81	119.19
961.87	24.79
966.03	3.31
968.50	26.22
985.31	28.64
989.92	14.02
1008.36	15.30
1022.71	14.64
1038.70	12.18
1050.72	26.48
1068.37	6.79
1083.64	47.11
1091.84	66.79
1125.45	82.03
1133.10	6.99
1155.60	15.80
1164.24	2.39
1171.22	23.35
1181.69	24.27
1204.65	12.02
1210.72	5.28
1242.68	159.36
1265.06	14.08
1275.93	59.52
1295.15	76.73
1305.51	67.74
1313.26	203.44
1320.27	163.75
1329.79	167.72
1361.59	134.95
1368.91	50.30
1372.21	124.53
1378.25	65.94
1393.62	35.87
1407.53	14.72
1418.42	68.23
1425.70	71.71
1429.43	92.98
1431.80	57.68
1439.90	43.24
1445.04	77.60
1450.79	105.27
1452.24	66.04
1458.56	13.56
1465.50	36.53
1471.08	69.40
1474.81	78.88
1597.34	142.73
1613.94	136.05
1618.76	89.21
1677.68	509.51
1714.09	498.16
2796.12	248.79
2952.00	238.23
2959.54	560.88
2966.44	238.50
2978.59	364.39
2984.05	588.12

2986.59	48.97
2990.52	362.31
2993.86	128.71
3013.47	75.55
3017.60	104.48

3028.35	173.30
3041.43	99.61
3046.12	60.22
3067.99	17.73
3068.21	61.06

3085.38	50.16
3089.11	83.42
3120.33	217.35
3451.29	134.42

Table S8. Solid-state DFT IR-active modes with frequencies (cm^{-1}) and infrared absorption intensities (km/mol) for DPA.

Frequency	IR Intensity
31.36	0.02
34.93	5.35
56.30	1.20
57.22	10.29
62.11	0.17
69.39	2.77
69.67	5.53
83.94	0.32
86.11	5.88
93.27	1.95
93.50	1.15
94.81	4.03
108.22	36.44
109.20	30.53
110.93	2.19
113.71	41.90
114.53	2.92
120.24	3.99
131.24	0.95
136.82	0.60
138.49	0.91
144.27	1.19
152.73	5.96
160.04	2.46
206.62	3.17
207.52	3.00
214.89	1.27
247.78	20.45
248.75	4.72
254.61	1.37
320.32	15.67
323.62	4.57
326.58	0.36
332.46	3.03
338.88	5.48
341.70	9.25
401.98	16.48
402.90	75.20
403.55	3.59
412.79	0.52
413.99	64.43
417.06	2.60
497.30	3.62
502.17	0.29
503.81	0.34
516.03	0.38
516.06	216.45
522.23	1.92
590.45	1.60
593.54	94.23
594.31	2.36
620.50	10.13
627.54	100.91
627.75	42.73
631.78	2.54
633.27	8.36
637.91	45.72
667.07	221.55
668.53	41.61
668.77	94.41
712.90	441.70
715.58	23.27
717.26	33.65
720.89	82.31
722.37	1.79
723.37	37.43
742.75	1257.90
743.41	177.39
746.03	445.37
757.39	22.26
759.44	0.60
766.76	8.36
813.87	71.46
815.30	44.11
818.10	17.61
832.01	45.43
832.91	34.70
838.30	54.89
855.85	0.96
856.46	64.96
862.80	0.32
875.24	133.04
891.03	11.86
912.99	549.52
915.86	0.01
916.89	113.20
918.79	0.11
919.76	1.53
920.04	315.72
921.26	141.87
943.75	0.38
944.57	32.79
944.92	7.91
951.57	0.65
955.25	1.25
958.02	0.24
963.29	1.67
964.23	0.59
966.85	17.16
977.23	256.89
978.22	10.51
978.96	7.63
988.13	0.68
992.54	150.05
993.20	19.93
1048.02	4.66
1051.02	62.59
1051.78	7.20
1053.61	10.96
1053.80	94.79
1055.71	0.26
1086.44	3.98
1093.94	220.96
1094.01	0.11
1100.07	8.07
1102.27	16.76

1103.55	57.54
1133.15	583.25
1134.19	1.52
1146.34	0.53
1165.30	48.39
1168.35	25.66
1169.85	0.95
1248.33	6.22
1249.01	96.60
1251.03	2.68
1266.48	16.37
1271.85	0.14
1277.43	2.66
1298.82	262.37
1301.13	61.83
1301.91	226.13
1305.51	113.32
1306.36	89.05
1309.20	8.92
1321.59	17.07
1322.15	3.25
1322.73	24.49
1364.56	52.95
1365.40	351.68
1365.54	739.48
1413.36	24.99
1414.45	20.75

1416.43	461.11
1430.75	4511.31
1433.22	917.49
1438.81	340.73
1462.13	1188.64
1463.81	299.50
1463.94	1864.24
1474.64	23.30
1475.64	1415.71
1482.99	488.82
1532.49	755.99
1536.18	6.60
1540.95	96.42
1563.60	177.44
1564.02	364.43
1564.42	12.41
1588.28	1420.93
1590.97	2291.46
1591.84	508.88
1602.94	303.80
1604.33	254.15
1605.17	30.95
1626.64	445.81
1627.71	1799.97
1648.56	160.20
2945.84	229.56
2969.26	6025.52

2982.25	10438.90
3076.94	17.24
3077.06	0.78
3077.07	78.96
3084.78	119.55
3084.87	9.67
3085.40	60.90
3093.35	196.98
3094.60	127.45
3094.80	3.78
3115.41	1.87
3115.65	44.70
3115.98	0.94
3135.22	0.02
3135.84	61.81
3136.37	37.74
3141.00	22.84
3141.36	34.28
3141.94	1.52
3143.32	0.95
3143.34	0.13
3143.48	5.50
3178.40	7.06
3178.44	0.80
3178.58	13.17

Table S9. Solid-state DFT Raman-active modes with frequencies (cm^{-1}) and relative Raman scattering intensities (normalized to 1000) for DPA at 78 K.

Frequency	Raman Intensity
26.41	736.73
28.12	370.50
36.54	37.73
46.87	92.53
48.78	197.12
49.76	85.51
53.37	1000.00
53.46	34.09
54.17	255.46
60.17	231.14
61.87	50.13
72.43	104.11
75.52	223.19
76.99	19.13
77.83	215.07
82.89	419.19
86.42	42.83
91.95	197.55
101.08	26.89
105.10	248.97
108.18	89.42
110.81	21.31
112.71	209.34
113.37	117.81
116.88	101.25
117.95	9.66
119.14	48.22
123.36	7.96
132.30	489.17
135.68	74.95
136.16	149.21
136.49	18.92
140.77	146.99
143.86	25.25
149.01	88.01
154.62	78.87
203.32	20.58
204.69	6.06
209.90	158.08
211.53	114.54
246.83	6.41
248.55	6.67
252.07	6.55
257.36	8.94
320.81	1.82
322.88	17.25
323.20	140.78
325.26	39.21
333.41	50.36
333.84	9.24
341.32	2.55
341.42	14.26
400.02	2.02
401.49	2.03
407.66	0.84
407.80	2.57
413.26	17.22
413.57	14.19
416.07	6.07
417.33	2.10
496.55	29.49
497.62	0.03
500.10	0.00
504.62	1.06
515.11	0.28
515.28	2.62
519.35	7.98
525.62	2.44
590.39	14.77
590.51	18.68
594.58	1.64
595.36	0.00
618.09	57.30
619.91	14.21
627.49	0.68
628.12	4.68
631.36	31.28
633.85	3.64
634.08	0.53
636.14	0.01
667.54	3.23
668.38	14.93
668.77	22.43
672.12	39.05
716.48	0.97
716.94	1.37
719.22	0.22
719.73	2.83
720.29	0.32
721.73	2.54
722.26	11.45
723.32	0.02
744.97	2.85
748.08	80.18
749.90	2.72
752.88	3.06
754.63	11.16
758.79	0.13
761.75	1.95
763.25	0.33
813.56	295.60
817.04	47.36
817.79	0.05
819.97	0.62
830.76	0.17
832.10	0.38
834.68	14.22
838.88	61.07
849.70	0.29
855.11	33.66
857.20	0.30
857.74	5.18
875.73	18.05
891.08	6.75
913.54	39.10

915.65	0.34
916.20	2.29
916.85	0.55
918.96	12.23
920.37	8.96
921.74	13.41
922.13	0.00
923.17	0.15
942.73	0.90
943.91	13.99
944.62	11.97
946.29	2.37
947.66	0.00
952.57	23.67
954.87	0.12
955.13	11.44
960.02	0.02
962.67	0.62
962.98	23.20
963.18	0.01
965.53	0.06
978.42	5.13
978.44	444.74
978.68	16.82
979.11	25.28
987.91	35.98
988.79	500.31
993.02	18.67
993.62	12.18
1048.31	2.66
1050.24	9.50
1050.28	17.35
1051.58	13.86
1052.60	9.99
1053.60	0.27
1054.56	0.31
1056.21	752.50
1084.82	2.31
1089.05	105.34
1093.01	8.33
1095.95	1.79

1099.76	0.05
1102.32	25.58
1103.02	0.00
1104.05	139.39
1133.42	0.75
1135.02	3.14
1143.03	3.69
1147.37	206.95
1166.47	1.85
1166.62	0.91
1166.98	20.02
1168.08	0.01
1248.49	0.21
1249.66	0.78
1249.96	6.38
1251.35	76.48
1267.21	343.34
1269.24	72.39
1272.75	5.53
1274.69	21.78
1299.60	6.18
1300.86	34.01
1301.27	70.16
1301.52	234.74
1304.48	1.31
1304.99	38.65
1306.04	3.31
1306.19	82.70
1319.24	0.13
1320.51	113.85
1321.84	9.77
1322.82	4.06
1359.93	2.22
1365.14	142.81
1365.91	6.58
1366.56	5.96
1412.71	103.46
1413.45	4.77
1415.34	33.18
1416.86	10.02
1430.75	0.39

1431.02	37.05
1431.97	5.55
1437.76	2.24
1458.11	18.15
1458.73	1.94
1461.55	5.88
1467.28	3.92
1474.91	6.56
1475.27	0.82
1476.50	5.50
1476.94	1.61
1533.05	1.80
1533.28	31.56
1538.22	56.69
1540.04	22.90
1563.01	4.55
1563.96	56.52
1564.39	3.75
1564.71	21.41
1584.43	122.11
1586.88	6.47
1588.36	1.93
1588.60	70.77
1601.45	408.10
1602.61	48.93
1606.01	513.29
1606.82	115.45
1628.04	2.52
1630.27	3.37
1646.41	0.90
1647.87	27.22
2944.13	10.26
2944.84	534.76
2975.54	17.69
2983.87	0.05
3076.32	14.75
3076.70	20.21
3076.95	125.48
3077.02	1.10
3084.61	72.59
3084.99	4.07

3085.12	3.78
3085.39	176.59
3092.81	0.46
3092.92	169.31
3094.14	15.06
3094.30	149.47
3115.19	67.93
3115.60	25.91
3115.69	142.86

3115.78	3.32
3136.07	0.43
3136.15	11.64
3136.79	12.90
3137.03	169.82
3141.10	17.92
3141.33	12.48
3142.77	61.42
3143.17	586.02

3143.30	3.02
3143.33	0.52
3143.41	3.05
3143.55	93.76
3179.20	175.71
3179.22	0.30
3179.33	25.70
3179.76	0.08

Table S10. Solid-state DFT IR-active modes with frequencies (cm^{-1}) and infrared absorption intensities (km/mol) for MPA:DPA.

Frequency	IR Intensity
24.19	0.27
26.95	0.00
35.77	0.67
36.77	2.70
44.22	0.03
45.32	3.92
49.13	20.51
51.19	0.81
51.37	0.59
54.76	0.09
56.44	0.41
62.34	1.89
63.45	3.54
65.31	3.19
65.62	0.24
71.72	17.80
73.68	8.76
74.00	1.46
74.68	0.13
79.24	2.04
80.54	3.93
82.30	4.78
86.72	3.34
87.18	0.44
90.09	8.90
92.31	0.90
93.98	1.75
96.23	0.85
96.81	3.53
98.55	30.92
101.19	16.34
105.64	39.72
106.44	2.81
107.48	5.32
108.41	0.35
119.28	3.13
121.48	3.49
129.96	11.48
135.53	26.86
137.89	7.13
138.36	14.50
145.54	1.20
147.73	0.25
153.89	4.52
153.98	7.07
159.49	18.22
159.75	4.26
168.96	8.75
173.06	48.73
177.24	10.46
177.71	84.06
180.83	7.17
182.19	2.34
185.42	7.66
187.11	2.23
191.92	72.01
194.62	16.62
197.69	5.91
199.38	20.16
215.90	13.97
221.49	6.12
225.43	3.13
225.73	66.39
234.24	100.25
236.11	30.03
238.01	5.90
238.25	14.08
252.51	1.21
252.54	0.02
255.98	25.22
256.98	48.21
287.17	22.79
287.18	4.39
319.21	5.71
320.22	5.43
329.09	6.01
329.45	21.55
331.61	13.31
331.72	1.08
343.90	9.05
344.11	12.42
354.12	1.07
354.42	77.36
357.40	58.76
357.80	38.23
366.23	1.85
366.36	6.20
402.39	10.99
402.99	3.34
404.21	0.00
404.48	36.43
408.36	4.03
408.40	42.62
430.95	2.78
431.32	0.80
452.97	11.07
455.65	272.28
463.98	142.54
464.87	44.83
490.39	0.03
490.84	4.84
499.98	0.73
500.26	0.02
515.04	92.55
515.41	11.74
517.88	62.23
521.07	1.88
543.94	19.73
544.36	72.71
552.24	17.01
552.49	0.36
567.41	16.72
567.84	6.29
593.40	14.25
593.46	26.84
605.62	2.07

605.68	29.35
630.11	109.24
630.91	0.27
633.22	23.44
633.48	1.57
642.68	132.43
644.17	5.75
647.32	244.89
648.01	3.51
673.59	1.27
673.94	173.92
676.66	47.32
677.43	393.03
680.84	36.31
681.32	117.87
690.32	2.75
690.43	32.70
720.58	19.11
721.15	9.55
722.05	36.36
722.41	1.65
727.27	41.24
727.80	9.85
733.37	158.69
733.39	5.26
744.14	48.82
744.21	1.09
754.65	581.96
756.81	7.77
768.83	215.13
769.81	9.05
778.41	72.22
779.44	12.11
808.76	38.25
809.06	87.85
816.22	103.84
816.27	164.53
835.11	72.07
836.06	45.78
837.12	25.41
837.81	64.64

855.95	292.97
856.11	292.12
862.29	182.51
863.09	31.43
876.39	2.88
876.55	1.21
908.01	45.60
908.68	54.64
916.46	192.05
916.87	57.27
920.11	10.39
921.05	58.69
931.23	0.44
931.23	13.03
944.20	178.81
945.54	24.85
947.57	24.20
947.92	33.73
950.36	12.31
950.86	16.92
962.01	240.11
962.60	208.86
964.51	60.82
964.96	5.54
968.93	19.06
969.41	4.46
980.28	3.75
980.30	54.25
980.72	130.12
981.84	38.83
985.55	0.20
985.75	12.38
989.61	1.32
990.05	34.24
1005.45	313.99
1005.99	472.18
1008.13	167.09
1008.97	0.41
1016.75	211.80
1017.32	31.85
1021.82	41.39

1022.24	13.38
1028.33	32.60
1028.55	1.52
1047.69	71.55
1047.87	79.02
1049.24	2.03
1049.28	256.50
1059.03	692.47
1064.60	448.70
1083.53	250.79
1084.13	0.12
1090.34	76.98
1090.79	10.85
1092.46	493.40
1093.75	27.67
1103.90	21.32
1104.19	70.12
1121.10	193.39
1123.50	470.90
1133.41	49.37
1137.38	0.46
1148.05	19.46
1148.66	252.17
1149.88	11.60
1150.79	218.48
1162.35	19.35
1163.11	1.14
1166.56	501.59
1166.92	163.45
1168.50	5.53
1170.17	200.56
1174.95	797.21
1175.97	130.06
1180.37	115.15
1182.34	67.40
1201.43	227.37
1207.14	22.58
1211.50	8.77
1214.89	286.75
1229.44	1099.23
1232.00	73.28

1249.32	266.20
1249.46	77.72
1257.08	642.85
1260.33	386.87
1267.12	85.65
1270.68	339.44
1279.41	12.78
1282.10	319.51
1297.51	38.02
1298.98	42.22
1302.29	6.54
1302.71	7.12
1308.82	5.95
1309.17	152.33
1310.25	133.94
1310.93	93.51
1314.16	419.88
1314.83	325.94
1319.52	11.34
1320.82	161.95
1323.94	11.82
1324.71	170.63
1344.86	3.09
1345.33	657.55
1354.72	1025.25
1356.20	2.09
1362.56	721.93
1362.74	191.05
1365.94	16.13
1365.99	14.52
1373.58	114.23
1374.07	69.28
1381.18	106.91
1381.40	10.58
1393.79	83.90
1393.85	613.73
1412.14	368.35
1412.72	287.09
1415.30	634.94
1418.26	84.22
1422.54	486.98

1424.00	5.47
1424.85	1191.80
1427.44	141.10
1428.97	44.31
1430.15	3.27
1432.36	269.80
1433.21	888.21
1435.85	359.27
1437.75	52.29
1439.40	27.84
1439.81	152.46
1442.30	39.61
1442.72	19.22
1443.17	101.57
1444.39	49.18
1446.71	115.11
1447.67	0.06
1447.76	70.22
1450.13	37.49
1457.01	39.44
1457.40	674.90
1462.26	33.95
1463.01	15.50
1474.57	889.50
1474.85	67.84
1475.25	40.32
1476.20	147.04
1527.61	140.03
1528.34	267.76
1556.19	140.72
1556.33	296.86
1561.58	814.42
1561.88	448.66
1590.58	2240.11
1591.84	67.53
1600.49	22.36
1601.66	25.91
1609.73	989.99
1610.15	94.38
1611.41	333.29
1612.49	99.50

1628.26	2197.56
1628.90	1332.06
1637.98	168.94
1638.01	1702.10
1678.73	1.53
1678.76	17.23
1711.82	4068.32
1717.61	3.39
1870.60	115.46
1874.30	15914.23
2958.91	56.97
2958.96	11.54
2962.21	87.53
2962.44	30.75
2963.18	248.61
2963.20	27.47
2972.31	49.40
2972.43	11.93
2975.71	6.46
2975.72	242.73
2978.90	11.74
2978.92	34.62
2979.53	245.09
2979.72	63.31
3014.04	44.43
3014.11	2.32
3022.01	0.87
3022.20	48.64
3023.67	87.66
3023.70	50.14
3039.16	1.08
3039.23	4.44
3054.02	0.17
3054.11	11.74
3058.65	2.57
3058.66	84.21
3065.57	7.30
3065.70	16.55
3066.73	47.94
3066.88	11.30
3073.95	63.44

3073.97	11.75
3082.66	45.54
3082.74	51.54
3084.46	25.95
3084.63	150.68
3086.00	49.27
3086.06	19.91
3106.48	81.24
3106.59	54.23

3119.68	138.48
3119.69	126.41
3127.12	42.42
3127.16	140.48
3133.50	284.32
3133.57	28.24
3136.23	26.45
3136.28	15.40
3141.72	0.01

3142.58	5641.79
3150.88	2.38
3151.03	24.23
3171.18	14.04
3171.25	37.29
3482.74	1550.26
3483.61	202.52

Table S11. Solid-state DFT Raman-active modes with frequencies (cm^{-1}) and relative Raman scattering intensities (normalized to 1000) for MPA:DPA at 78 K.

Frequency	Raman Intensity
15.46	58.10
21.78	85.13
32.20	889.31
36.58	52.78
37.09	247.69
39.00	444.70
44.72	80.66
45.26	647.89
50.29	199.28
50.60	169.46
51.60	38.15
53.14	99.40
55.85	104.38
59.07	1000.00
59.43	287.98
59.45	32.03
60.11	22.50
66.40	164.23
69.29	530.09
71.29	39.84
74.40	33.15
76.64	349.43
79.24	364.23
79.99	402.98
81.37	62.37
86.23	50.04
86.88	57.05
89.38	182.50
92.95	30.72
94.30	440.76
94.99	12.51
98.41	90.91
98.97	256.03
101.86	244.15
102.19	128.83
106.93	85.51
107.27	16.40
110.13	55.18
118.83	48.42
120.23	4.13
130.07	128.59
132.50	97.74
134.72	28.58
139.80	85.87
145.24	32.70
146.20	31.76
154.24	65.12
155.57	22.62
157.91	8.19
158.05	44.00
171.22	28.56
173.45	43.88
178.15	4.82
178.16	7.55
180.19	1.57
182.34	81.89
185.03	9.41
185.50	24.34
193.38	32.32
193.56	90.62
197.57	118.87
199.78	3.34
217.69	9.92
219.82	26.45
222.05	63.38
223.68	14.46
232.28	6.36
235.43	32.63
238.45	24.53
238.74	46.60
251.90	181.05
252.77	0.97
256.06	14.38
256.48	15.41
287.06	9.76
287.43	0.66
319.76	7.62
320.47	46.09
328.36	54.05
328.85	56.64
332.05	41.87
332.43	123.43
343.34	49.99
343.98	0.84
353.90	50.26
354.60	29.17
357.46	14.67
358.39	47.06
365.63	67.72
366.21	11.91
402.39	5.70
403.07	21.87
404.60	15.62
405.34	20.81
408.21	13.35
408.81	1.23
430.88	17.08
431.65	11.59
452.72	38.53
454.78	47.76
463.44	105.06
465.07	1.23
489.85	3.00
490.88	73.57
500.04	7.53
500.57	3.11
514.64	40.52
515.21	5.17
519.32	6.06
519.57	14.42
543.15	182.75
545.56	10.77
552.49	1.11
552.59	42.28
567.04	4.83

567.94	53.03
594.03	16.80
594.08	38.19
605.42	2.19
605.63	71.64
630.51	28.06
630.88	6.20
632.97	3.73
633.23	21.21
643.52	4.42
644.51	0.29
646.74	43.76
647.62	61.82
673.08	152.17
673.21	3.70
675.33	6.24
678.17	1.77
680.24	1.93
680.89	1.91
689.77	6.88
690.74	0.02
719.45	1.63
719.50	27.03
721.42	9.81
721.67	1.43
727.82	4.07
728.29	151.43
733.70	15.28
733.98	1.58
744.07	6.53
744.14	82.27
756.69	36.91
757.29	10.21
769.87	3.23
771.15	8.30
778.64	3.90
778.66	43.46
808.01	7.18
808.68	40.90
816.54	127.40
817.23	5.53

834.50	10.51
835.23	2.51
837.46	273.25
837.60	3.87
852.06	1.40
852.27	17.41
860.92	3.61
862.58	12.33
876.16	2.99
876.76	7.27
907.36	37.17
908.13	18.51
917.33	1.54
917.78	8.46
919.82	9.05
920.74	14.69
930.51	14.82
930.65	14.66
944.64	0.82
944.85	21.25
949.11	1.35
949.33	12.43
950.83	9.52
951.41	13.53
961.83	46.21
963.17	10.58
965.89	5.60
966.19	8.29
969.05	4.24
969.78	1.71
979.81	18.26
980.61	15.58
980.87	3.26
981.08	363.33
984.84	47.83
985.25	6.77
989.58	19.84
990.61	30.30
1005.04	25.57
1006.42	17.94
1007.49	7.43

1008.19	415.91
1017.08	0.33
1017.16	4.23
1021.79	2.26
1022.44	2.28
1028.07	9.24
1028.82	2.20
1048.63	5.22
1048.93	348.91
1050.21	144.57
1050.48	5.85
1058.61	12.38
1063.21	1.73
1083.56	4.82
1084.30	4.93
1090.38	45.75
1090.90	75.51
1092.83	0.44
1093.53	38.58
1103.54	37.41
1103.81	18.15
1121.31	37.97
1123.61	14.33
1133.14	1.85
1137.22	10.62
1147.96	18.72
1148.52	21.74
1149.57	31.68
1150.86	23.83
1161.44	11.43
1165.01	15.20
1165.80	15.91
1168.45	13.20
1170.04	3.23
1170.10	11.03
1174.56	12.36
1177.91	11.86
1180.95	30.04
1182.23	72.13
1201.80	22.04
1206.36	9.13

1207.48	3.19
1215.00	30.94
1230.21	31.42
1232.03	46.24
1250.74	15.59
1250.85	16.02
1258.02	6.99
1258.57	204.96
1265.11	17.86
1268.23	5.87
1279.93	124.97
1280.90	7.40
1297.86	0.43
1298.49	112.41
1302.78	43.44
1303.14	327.33
1307.87	3.51
1308.60	5.68
1310.23	9.78
1311.15	23.40
1316.42	43.75
1316.57	312.62
1319.88	29.70
1321.25	1.47
1323.80	18.98
1324.70	48.57
1343.97	7.26
1345.06	64.44
1354.35	3.93
1354.85	46.31
1362.41	0.15
1363.02	218.68
1365.73	95.46
1366.98	29.57
1373.33	93.31
1374.17	36.36
1381.25	25.56
1381.62	90.80
1393.69	4.03
1394.42	26.18
1412.58	165.49

1414.37	16.61
1417.66	51.29
1418.76	72.25
1423.69	18.08
1424.31	16.38
1427.24	10.41
1428.72	20.18
1429.93	32.60
1430.96	11.47
1431.56	16.29
1432.71	4.29
1436.10	29.23
1437.68	0.79
1439.08	25.38
1439.53	9.06
1442.15	4.65
1442.72	18.37
1444.19	41.38
1444.43	29.33
1445.35	62.66
1447.55	17.50
1448.40	41.16
1448.83	28.81
1455.28	10.34
1455.58	17.63
1462.03	53.95
1462.73	0.09
1474.21	6.40
1475.27	7.12
1477.42	22.04
1477.96	0.09
1528.37	105.05
1529.25	3.87
1555.86	6.84
1556.45	7.58
1562.38	135.46
1562.53	49.15
1592.00	40.31
1593.34	60.27
1601.41	729.66
1601.45	34.01

1610.29	34.56
1610.45	13.51
1610.97	18.77
1611.06	119.79
1628.22	235.95
1630.26	0.85
1640.16	60.24
1643.75	5.63
1678.84	347.27
1678.87	30.50
1705.58	480.88
1708.26	66.92
1862.79	21.57
1863.79	220.00
2958.80	167.34
2959.08	10.20
2962.70	3.50
2962.78	68.67
2962.98	23.56
2962.99	636.02
2972.44	165.55
2972.80	32.81
2975.59	488.93
2975.61	6.30
2978.86	324.96
2978.97	31.67
2979.57	304.65
2979.74	6.86
3013.99	171.35
3014.00	23.28
3021.94	92.35
3022.22	26.35
3023.59	139.20
3023.67	18.38
3039.11	189.68
3039.15	33.01
3053.70	14.44
3054.07	50.02
3058.59	24.96
3058.66	4.40
3065.66	28.43

3065.74	17.94
3066.64	63.70
3066.71	28.49
3073.83	45.88
3074.12	39.21
3082.36	3.31
3082.69	8.88
3084.66	139.33
3084.82	13.08
3085.94	59.51

3085.99	44.69
3106.54	51.81
3106.59	84.78
3119.59	156.70
3119.61	73.01
3126.49	115.79
3127.09	61.84
3132.14	23.71
3132.33	18.07
3135.92	154.80

3135.96	32.62
3140.45	2.23
3140.93	426.04
3150.62	232.94
3150.95	1.75
3171.08	86.23
3171.23	6.75
3482.86	106.37
3483.04	2.97

Table S12. Solid-state DFT IR-active modes with frequencies (cm^{-1}) and infrared absorption intensities (km/mol) for MMF.

Frequency	IR Intensity
18.90	0.98
30.41	0.10
31.04	0.44
34.01	0.59
36.53	0.46
45.43	1.56
46.02	0.89
47.60	0.65
52.45	0.07
53.60	0.11
60.86	0.59
63.42	17.93
64.60	6.03
69.82	1.57
75.23	0.98
75.81	7.24
77.89	3.12
83.20	6.27
84.17	1.55
90.35	9.33
90.53	0.00
93.68	24.53
94.86	1.60
99.94	0.79
100.13	3.02
106.78	5.60
106.82	1.51
109.36	7.74
114.19	11.51
115.89	26.28
123.47	7.30
124.79	14.92
128.44	47.40
134.08	11.76
136.22	9.74
142.84	10.67
145.02	3.49
146.99	12.20
148.07	0.26
150.39	10.01
152.76	7.87
156.16	1.06
159.56	47.03
168.14	0.76
169.03	11.11
171.38	0.00
176.24	14.34
193.26	10.90
195.19	6.13
200.49	2.01
201.77	1.25
204.34	0.46
206.77	16.23
215.71	48.06
224.21	0.07
226.42	29.27
228.45	9.89
238.15	15.05
242.33	50.96
256.00	0.29
256.63	26.65
267.30	26.71
269.34	4.41
275.16	14.14
277.26	13.05
277.90	12.24
280.56	4.91
295.07	21.24
296.78	0.15
301.19	61.81
301.59	1.36
332.90	73.60
333.83	24.48
343.91	36.30
345.82	48.32
363.12	27.16
364.79	4.76
364.97	102.52
367.47	133.61
368.85	4.93
371.37	7.11
394.55	63.71
395.65	14.39
404.28	11.55
404.49	6.64
418.73	1.17
419.33	2.02
432.74	17.65
433.35	2.09
445.49	54.79
446.22	19.84
460.26	8.10
460.60	7.32
472.91	29.45
473.45	14.84
487.25	0.11
489.46	2.84
522.40	5.72
522.58	9.55
531.28	59.11
531.43	7.63
538.63	60.66
539.09	7.95
561.60	9.12
562.19	16.76
570.88	45.64
574.04	0.05
576.42	4.47
579.02	12.32
624.83	25.98
625.15	20.21
633.98	5.61
636.75	26.26
657.28	81.43
658.81	24.69
680.51	42.72

681.55	0.22
692.39	47.62
692.66	5.60
719.60	0.28
719.65	52.08
727.01	3.88
727.48	2.97
733.39	26.01
733.81	102.23
742.43	6.09
744.86	5.69
782.37	105.95
782.83	152.63
784.28	149.72
788.06	641.39
795.46	7.46
795.67	38.83
797.63	39.29
799.57	11.53
825.50	215.67
827.35	165.33
840.73	2.63
841.51	0.65
846.13	65.00
846.31	190.52
852.27	215.47
852.34	6.44
895.45	8.39
896.75	169.58
905.70	104.48
906.21	5.57
915.26	2.30
915.61	25.05
943.41	61.60
944.64	265.15
955.16	40.06
955.48	444.09
962.37	7.38
962.49	40.77
970.04	16.69
978.08	16.02

981.29	0.00
983.23	86.28
986.12	143.63
988.04	6.73
993.26	0.20
993.35	109.63
996.76	36.31
998.26	337.36
1012.42	871.67
1017.41	45.85
1024.71	23.40
1027.18	36.92
1027.27	12.84
1032.27	0.09
1039.33	63.10
1041.94	5.08
1046.71	1.92
1046.89	29.71
1050.10	13.07
1052.61	135.32
1053.01	859.54
1053.70	12.63
1064.86	69.60
1065.42	279.15
1077.33	5.66
1077.36	18.36
1086.04	9.50
1086.56	252.90
1088.52	94.01
1088.71	156.21
1097.73	298.37
1097.90	704.27
1120.24	0.35
1121.95	41.03
1123.72	3.25
1124.47	47.00
1127.55	223.57
1128.84	106.24
1130.71	95.03
1132.96	2043.34
1139.14	186.98

1140.84	44.69
1156.69	340.51
1157.84	204.31
1162.66	12.02
1162.71	0.15
1164.10	24.07
1166.81	188.63
1173.99	197.77
1174.69	29.53
1177.72	162.68
1179.47	25.60
1184.99	898.57
1188.41	641.16
1203.72	38.06
1205.23	114.76
1227.00	94.72
1228.90	27.84
1237.55	0.89
1238.03	22.55
1255.42	85.55
1256.50	32.67
1263.00	219.62
1264.14	5.39
1270.95	13.39
1271.02	80.75
1277.94	19.03
1279.48	151.46
1282.91	205.61
1283.91	221.83
1295.57	65.07
1295.59	14.85
1298.00	308.86
1298.57	222.02
1306.21	39.45
1306.74	1.36
1313.87	950.00
1314.07	21.66
1315.62	40.30
1319.13	92.14
1324.33	18.50
1324.45	4.92

1327.04	35.48
1327.86	3.23
1333.45	88.56
1333.86	7.63
1349.29	2.49
1350.29	27.23
1363.76	10.10
1368.63	53.29
1369.39	4.65
1370.19	81.39
1371.38	19.34
1371.70	8.72
1371.90	34.55
1373.45	3.50
1376.62	111.16
1376.80	0.24
1381.97	137.01
1383.14	42.17
1386.84	238.07
1391.91	131.14
1393.58	3.72
1393.78	30.82
1410.10	246.54
1411.20	139.48
1414.06	13.36
1414.08	37.50
1419.62	69.74
1420.40	8.12
1424.19	102.73
1424.71	39.78
1425.70	58.36
1428.52	200.53
1431.33	3.19
1431.67	105.10
1432.32	3.41
1434.28	230.42
1435.12	104.67
1435.32	8.71
1437.11	153.55
1437.89	240.12
1439.88	94.38

1441.24	4.29
1441.88	13.56
1443.85	0.94
1444.74	78.12
1445.41	13.81
1445.56	11.79
1446.55	13.02
1452.34	43.23
1452.41	60.43
1459.98	79.62
1460.66	35.30
1461.18	82.96
1462.43	7.01
1463.37	9.90
1466.31	0.93
1471.89	198.26
1473.11	0.36
1584.81	229.80
1586.56	607.78
1610.81	189.33
1611.37	553.18
1669.41	45.98
1670.08	2.76
1700.04	2441.55
1713.19	2026.32
1714.66	355.91
1722.11	1992.62
2832.34	264.85
2833.03	159.00
2849.44	454.16
2850.33	27.99
2867.19	278.78
2867.25	148.17
2930.57	418.85
2931.35	28.07
2946.83	24.37
2946.96	57.36
2954.70	40.83
2954.94	220.05
2956.14	108.02
2956.26	263.56

2968.61	4.47
2969.66	30.20
2976.34	1.71
2976.34	148.31
2976.40	43.73
2976.41	0.28
2979.82	47.18
2980.27	52.27
2983.05	90.41
2983.43	12.34
2988.27	13.47
2989.74	28.03
3003.72	0.57
3003.80	32.97
3006.13	9.42
3006.20	32.09
3011.55	47.81
3011.61	1.31
3013.42	40.18
3013.45	1.81
3020.72	15.25
3021.02	13.36
3023.77	91.70
3024.11	73.14
3033.43	5.33
3033.56	10.57
3036.98	14.74
3037.80	129.72
3040.65	12.52
3040.77	83.51
3041.04	9.27
3041.81	0.87
3051.38	0.00
3051.70	94.17
3057.75	11.08
3057.92	4.57
3071.07	1.77
3071.30	20.94
3087.63	13.59
3087.65	4.47
3089.25	39.47

3089.65	20.76
3089.92	29.86
3089.97	4.09

3106.36	58.82
3106.44	7.54
3266.90	2593.85

3268.90	3453.17

Table S13 Solid-state DFT Raman-active modes with frequencies (cm^{-1}) and relative Raman scattering intensities (normalized to 1000) for MMF at 78 K.

Frequency	Raman Intensity
21.72	143.83
25.29	38.06
31.76	210.67
34.54	30.11
37.31	48.65
40.03	58.38
40.94	2.01
45.20	37.81
46.43	14.52
48.98	14.93
49.55	102.18
56.58	118.41
64.35	36.91
65.85	47.54
68.22	69.38
69.53	310.40
74.05	72.31
76.13	17.60
84.44	10.51
84.97	87.09
86.26	146.20
88.95	74.49
90.80	127.49
93.11	197.22
95.68	53.52
99.48	26.30
100.99	17.32
104.03	63.84
111.13	33.27
111.27	9.70
112.99	220.93
113.69	29.62
118.96	49.99
121.84	340.75
122.55	163.37
133.06	69.77
133.10	24.41
136.74	19.83
144.61	17.69
144.66	25.79
146.59	36.17
147.64	21.07
155.75	40.17
158.47	49.80
159.59	36.29
159.97	68.51
169.92	8.39
172.74	51.42
174.01	0.70
176.15	51.90
191.26	16.96
191.47	0.74
197.80	0.94
200.20	11.66
202.33	47.85
208.51	20.31
215.97	9.66
219.68	83.21
228.06	0.63
228.61	23.07
240.55	86.60
240.83	17.30
257.11	22.99
258.75	7.74
261.82	21.97
266.25	3.18
275.63	5.56
276.96	96.28
278.06	31.43
278.64	33.41
295.77	12.07
296.19	1.24
296.83	21.74
299.40	9.29
331.93	32.85
333.73	20.14
343.53	15.91
345.52	8.65
363.84	40.96
364.88	3.21
365.34	23.29
368.53	20.49
369.90	15.41
371.07	2.19
393.21	2.07
395.32	0.19
403.24	49.45
405.59	29.03
418.42	12.68
419.45	31.31
432.53	113.70
433.42	20.00
445.19	20.22
446.58	3.27
458.61	23.48
461.66	48.09
472.82	41.96
474.35	98.72
487.51	18.04
489.19	15.79
520.04	14.03
522.62	139.89
530.20	21.27
530.30	9.64
538.46	24.79
538.51	184.50
557.77	9.64
562.15	14.75
570.03	3.02
574.42	115.54
577.70	4.75
577.76	14.94
625.11	19.85
625.47	17.95
635.37	37.62

635.60	3.63
658.52	25.76
658.56	7.56
679.14	14.64
679.35	2.69
691.78	9.01
692.14	32.20
718.92	1.91
720.87	210.61
726.15	51.87
728.55	14.23
733.91	6.19
733.93	0.62
742.63	187.29
742.88	10.22
782.73	13.05
783.95	22.08
785.79	17.83
790.83	3.91
795.26	32.85
795.29	21.99
798.93	4.98
799.29	28.33
825.77	30.48
826.32	2.66
840.66	8.06
841.47	7.11
845.26	4.01
847.09	12.46
852.50	61.83
852.80	2.36
894.42	198.86
895.40	4.09
905.77	0.91
906.60	5.24
914.66	3.54
915.96	49.52
943.34	3.04
945.74	36.11
952.57	12.64
954.49	97.88

962.38	0.68
962.39	6.72
972.14	13.36
978.89	1.62
982.41	3.28
983.68	45.10
984.51	19.76
987.88	2.23
991.90	0.66
992.45	25.91
996.69	16.23
999.78	15.64
1013.26	24.86
1017.03	7.63
1024.78	4.12
1026.39	27.07
1028.06	5.45
1031.08	5.05
1038.76	1.19
1042.28	9.49
1045.69	25.08
1046.98	1.07
1050.16	0.30
1052.72	0.22
1056.12	21.61
1056.77	8.07
1067.14	9.00
1069.60	0.23
1077.06	12.69
1077.10	0.63
1086.05	34.65
1086.82	0.79
1088.09	33.44
1089.16	4.70
1095.40	60.29
1096.08	25.97
1119.52	41.31
1120.97	15.65
1122.45	7.19
1122.85	6.05
1127.16	51.53

1130.07	5.96
1134.48	1.55
1140.11	0.63
1140.54	53.34
1146.83	1.12
1156.91	13.99
1159.74	5.39
1161.71	2.67
1164.43	2.45
1165.53	6.76
1165.77	1.38
1173.54	48.61
1174.33	6.18
1179.05	24.47
1179.49	2.68
1184.87	21.04
1188.54	12.30
1203.71	30.78
1205.32	4.96
1226.58	35.12
1230.28	17.47
1236.24	1.90
1236.99	9.90
1255.12	16.77
1256.65	6.29
1263.67	42.99
1265.54	14.23
1270.67	32.99
1271.19	11.20
1278.41	50.99
1279.13	0.00
1283.18	18.93
1284.02	32.17
1296.86	18.42
1297.07	14.38
1298.35	195.21
1299.99	19.85
1305.54	39.40
1305.88	34.39
1314.71	72.85
1316.46	17.73

1317.01	8.59
1319.70	190.68
1322.63	91.87
1323.46	23.96
1327.76	3.74
1327.95	21.25
1332.73	63.70
1334.29	12.68
1350.47	6.27
1350.50	6.19
1363.31	4.88
1368.67	47.55
1368.73	8.83
1370.48	18.81
1371.41	52.66
1371.63	15.86
1372.48	55.76
1373.19	13.08
1377.42	27.67
1377.50	23.84
1381.59	61.27
1381.78	200.03
1385.99	23.75
1391.26	13.67
1393.08	16.89
1394.70	14.76
1410.66	11.91
1411.30	53.41
1412.72	12.34
1414.50	14.94
1421.82	28.06
1423.58	0.25
1424.74	56.79
1425.04	4.65
1427.96	72.06
1428.79	1.06
1429.35	79.39
1429.83	66.88
1430.25	12.21
1433.23	48.26
1433.51	16.63

1435.00	20.75
1436.78	1.31
1437.74	41.35
1440.12	38.37
1440.17	25.49
1441.51	10.78
1443.43	6.99
1445.51	50.10
1446.26	12.52
1448.34	6.19
1449.10	13.08
1452.38	29.90
1453.44	5.13
1459.92	40.99
1461.14	4.32
1461.53	6.56
1461.55	101.21
1463.58	46.82
1463.69	30.28
1471.32	5.06
1472.38	71.84
1584.85	112.56
1586.18	74.12
1610.31	175.76
1611.53	31.14
1669.26	356.08
1670.19	0.20
1701.28	593.44
1712.17	164.89
1715.45	118.08
1724.00	0.65
2832.37	75.15
2832.94	21.12
2849.59	154.57
2850.51	18.58
2867.03	328.22
2867.27	64.60
2930.55	137.50
2931.62	8.00
2946.59	217.52
2946.87	10.98

2954.70	698.84
2955.29	18.83
2956.21	85.27
2956.59	30.45
2967.96	15.30
2968.55	50.54
2976.35	127.92
2976.37	139.15
2976.68	12.06
2977.01	4.17
2979.85	240.44
2980.03	6.26
2983.06	275.75
2983.34	5.39
2988.16	99.24
2990.01	1000.00
3003.71	131.07
3003.86	30.27
3006.09	3.31
3006.15	11.78
3011.62	86.52
3011.63	37.55
3013.61	86.99
3013.70	51.66
3020.98	190.83
3021.11	42.54
3024.18	138.27
3024.18	0.46
3033.24	87.19
3033.47	26.70
3037.35	328.64
3038.71	94.79
3040.68	54.49
3040.71	46.01
3041.31	84.36
3041.51	34.13
3051.36	12.46
3051.42	51.90
3057.88	69.72
3057.89	7.31
3070.97	21.72

3071.27	100.39
3088.12	27.45
3088.76	15.74
3089.35	92.25

3089.76	6.23
3089.82	77.01
3090.00	1.86
3106.44	53.99

3106.50	59.97
3267.31	187.33
3269.46	66.52
